# Salt Effects on the Growth of Mixed Anionic and Zwitterionic Surfactant Micelles

**DOI:** 10.1021/acsomega.6c07682

**Published:** 2026-10-16

**Authors:** Michel Masella, Céline Houriez, Fabien Léonforté

**Affiliations:** † Laboratoire de Biologie Structurale et Radiobiologie, Service de Bioénergétique, Biologie Structurale et Mécanismes, Institut de Biologie et de Technologies de Saclay, CEA Saclay, F-91191 Gif sur Yvette Cedex, France; ‡ Centre for Energy Environment Processes (CEEP), Mines Paris, PSL University, 77300 Fontainebleau, France; § Research & Innovation, 6439 L’Oréal Group, 93600 Aulnay-Sous-Bois, France

## Abstract

We investigate the changes in 3:1 mixed sodium dodecyl sulfate (SDS) and cocamidopropyl betaine (CAPB) surfactant solutions (at the 400 mM scale) upon addition of alkali/halide salts up to a concentration of 2.5 M using coarse-grained molecular dynamics simulations coupled with a Hamiltonian replica exchange scheme and accounting for long-range electrostatic effects. Upon addition of salt, micelle properties (size, morphology, degree of neutralization, equilibrium between surfactant chains within ‘end-cap’ and ‘cylindrical’ micellar subdomains) as well as surfactant solution shear viscosities all obey a linear regime until a salt concentration of about 1 M. Then, they obey a new regime in which they all rapidly and nonlinearly diverge, in line with available experimental evidence regarding similar mixed anionic/zwitterionic surfactant solutions. Interestingly, the transition between these two regimes can be rationalized by considering an equilibrium between only SDS chains in ‘end-cap’ and ‘cylindrical’ micelle domains (according to the pseudophase thermodynamic framework), with CAPB chains taken into account implicitly. Although there is room for improvement, our simulation protocol appears to be a promising tool to investigate the effects of cosurfactants and/or hydrophobic (fragrance) molecules on surfactant solutions.

## Graphical abstract

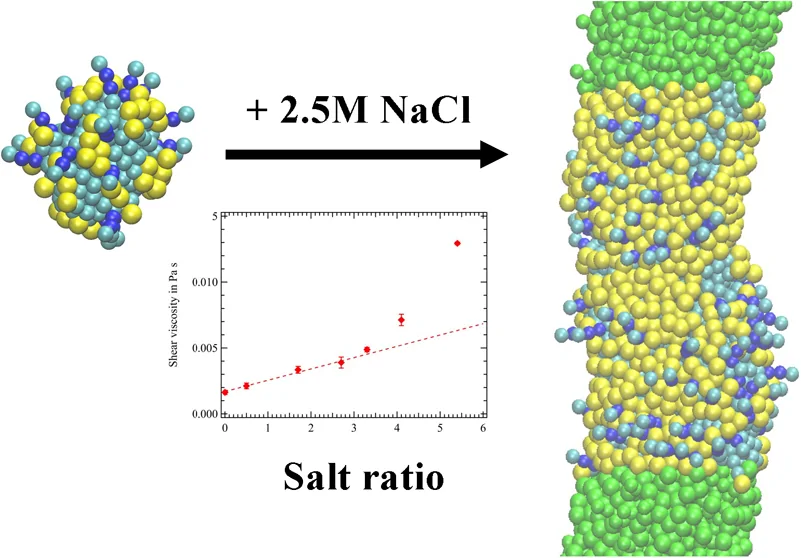

## 1 Introduction

In sufficiently concentrated aqueous solutions, surfactant molecules self-aggregate in nanostructures (micelles) with various sizes and geometries (from spheroidal to branched-like morphologies), which modulate the surfactant solution rheological properties, including their shear viscosity. Among all the surfactants investigated so far, the anionic sodium dodecyl sulfate (SDS) and the zwitterionic cocamidopropyl betaine (CAPB) are of particular interest because of the large amount of industrial applications, from petroleum processes^1^ to the cosmetic/personal care industry.^2^ We may also quote a recent study dealing with the relation between CAPB micelle geometries and skin irritation phenomena.^3^

Aside from physical conditions (in particular temperature^4,5^ ) and the surfactant *per se* (mainly its alkyl chain length^6,7^ ), micelle geometries are highly sensitive to surfactant concentrations and their chemical environment. For pure SDS solutions, surfactant self-aggregation in spheroidal micelles starts at relatively low critical micelle concentration (∼8 mM^8^). Increasing SDS concentration yields micelle shape transitions from spheroidal to elongated (denoted as the SE transition hereafter), rod-like, wormlike, and branched morphologies of increasing size.^8,9^

The presence of a zwitterionic cosurfactant like CAPB strongly (and nonlinearly) modulates the surfactant concentration *c*_surfactant_^SE^ at which the SE transition occurs. *c*_surfactant_^SE^ first appears to obey a sigmoid law with respect to the CAPB/SDS ratio: it decreases from 250 mM in pure SDS solutions toward values weaker than 50 mM for CAPB/SDS ratios ranging from 1:1 to 8:1 (whereas *c*_surfactant_^SE^ is ∼300 mM for pure CAPB solutions).^10^ Moreover mixed spheroidal SDS/CAPB micelles are larger in size compared to equivalent pure SDS or CAPB solutions, by about 30%, regardless of the surfactant ratio.^10^ Note, however, that the micelle sizes experimentally reported can vary up to a factor of 2 for a specific solution (see data from refs 11 and 12 regarding SDS solutions at the 200 mM scale with no added salt, for instance).

The above picture of micelle shape evolution relies on assuming compact micelle geometries stabilized by an overall large hydrophobic core (made of surfactant alkyl chains) and by peripheral strong electrostatic interaction networks among surfactant-charged moieties and counter/salt ions. Moreover, charge screening effects from salt cations interacting at a short range from micelle surfaces also favor micellar growth.^8,11,13–16^ However, more complex evolution schemes taking into account micelle swelling phenomena cannot be ignored.^9^ Overall, weak water insertion phenomena are reported within pure SDS micelles and they mostly correspond to insertion within the micelle external layer of anionic heads.^8,11^

A well-known effect arising from the addition of salt to an anionic surfactant solution (in the spheroidal concentration range) is the abrupt transition of the solution shear viscosity η̅^0^, which varies from the neat water value η̅_w_^0^ up to a peak that can be larger by several orders of magnitude than η̅_w_^0^, and then, it decreases as the amount of added salt further increases. These “salt curves” result from the charge screening of salt cations that favors the transition toward elongated and then wormlike micelles whose intricate network is responsible for the strong increase of η̅^0^. Salt curves are well documented for the sodium dodecyl ether sulfate, SLES_*n*_, a class of surfactants, which differ from SDS by the presence of *n* (usually 1–3) ether groups – O–CH_2_–CH_2_ in between the anionic head and the main alkyl chain. See the recent studies of refs 17–22 for solutions of SLES_1–2_ with alkali/halide added salts. For instance, the location of the viscosity peak is shifted to lower salt concentration *c*_salt_ and its height is lowered for chloride salts within the alkali series Li^+^ to Cs^+^.^20,23^ Moreover, the relative amount *X* of added NaCl compared to the total surfactant concentration *c*_surfactant_ to reach the viscosity peak decreases as *c*_surfactant_ increases. For instance, the viscosity peak for pure SLES_1_ solutions with added NaCl salt is located at *X* ≈ 3–4 for *c*_surfactant_ = 260 mM,^18,23^ whereas it is located at *X* ≈ 9–10 for *c*_surfactant_ = 160 mM.^20^ These data are in line with those of mixed 4:1 solutions of the palmitic sulfonated methyl ester and CAPB surfactants (at the 350–400 mM scale, the salt peak is located at *X* ≈ 2), as well as for solutions of pure sodium dodecyl benzene sulfate (at the 300 mM scale, *X* = 3.0 and 3.6 with added salts NaCl and LiCl, respectively)^24^ and for mixed 2:1 SLES_1_/CAPB solutions (at the 600 mM scale, *X* ≤ 2).^19^ Regarding the effects of NH_4_Cl and NH_4_Br salts on pure SDS solutions (at the 300 mM scale), we may mention a study showing the peak curve to be located at *X* ≈ 4,^25^ which is also in line with all the latter data.

As far as we know, only a few experimental studies provide viscosity data regarding SDS solutions, and these data do not allow us to provide a clear picture of their salt curves. For instance, Jamadagni et al.^26^ reported viscosity data that have not still reached a maximum for 3:1 and 2:1 SDS/CAPB solutions with the *c*_surfactant_ = 400 mM scale for added NaCl *c*_salt_ of up to 300 mM (in that study, CAPB is replaced with LAPB, for lauramidopropyl betaine). These data are in line with earlier shear viscosity values for 2:1 SDS/CAPB solutions at the 400 mM scale with 400 mM added NaCl: they are still close to neat water.^27^ The transferability of SLES_1_ data to SDS solutions is far from obvious as their properties can significantly differ.^28^ Nevertheless, micelle size data reported in ref 13 for pure SDS solutions at the 50 mM scale suggest the viscosity peak to be located at about *X* ≈ 10–12 for added NaCl. The above discussion thus suggests that the peak should be located within *X* = 2 and 5 for 300/400 mM SDS solutions. These *a priori* estimates hold for mixed 2:1 SDS/CAPB solutions at the 400 mM scale as their main features are close to SDS ones.^10^

Impressive new spectroscopy techniques are now available, including cryotomography electron microscopy (cryo-TEM), which provide direct insights regarding the equilibrium among micelle morphologies in salty solutions.^20,29^ Interestingly, for SLES_2_ solutions at the 200 mM scale, cryo-TEM data show the appearance of disklike micelles for concentrations of added salt KCl close to the solution viscosity peak. Disc-like micelles are rarely mentioned for SDS/SLES_*n*_-based solutions.^30^ However, they have been reported for a 4:1 SDS/CAPB solution in the presence of lauryl acid.^31^

However, the cryo-TEM resolution still prevents us from getting detailed insights into the phenomena at the sub-macromolecular scale. Molecular dynamics (MD) computer simulations allow one to complement experiments to better understand the effects of local microscopic interactions on micellar geometries (including SDS head charge screening by salt cations). MD simulations are based on numerical approaches taking explicitly into account all of the system atoms (all-atom simulations) or on approaches where sets of atoms are modeled as a single bead (coarse-grained, CG, simulations).

The atomic size of a molecular system corresponding to realistic aqueous micellar solutions is at least at the 10^5^ atom size scale and above,^32^ and the relaxation time of charged systems dissolved in aqueous solutions is at the μs scale and above. Although standard *all-atom* MD simulations are regularly reported (see among others refs 33–40 and the reference mentioned therein; these studies mostly focus on pure SDS solutions) CG approaches relying on the dissipative particle dynamics approach^37,39,41–47^ and on MARTINI force fields^48,49^ are the most efficient to simulate large molecular systems on such a time scale. Because of the softness of CG interaction potentials, the MARTINI approach allows the use of large MD time steps that are 5–20 times larger than for standard *all-atom* force fields. For instance, we may quote here recent MARTINI-based MD simulations at the 2 μs scale of SDS/CAPB solutions comprising 128,000 water beads (that corresponds to 512k real water molecules) performed by Zhou and Ranjith.^32^

The standard MARTINI approach relies on truncating both the Lennard-Jones and Coulombic potentials for distances larger than 11 Å. Using earlier MARTINI force field versions than the most recent 3.0b,^49^ this assumption is reported to yield artifacts in modeling ionic surfactant solutions (such as unphysically large surfactant aggregating phenomena). For a more realistic modeling of ionic surfactant solutions, MD simulation studies^50,51^ show the need to account for long-range electrostatic interactions and to perform simulations at the 6 μs scale and above. In these simulations, water was implicitly taken into account (using recalibrated MARTINI parameters^50^) and the size of the beads modeling the surfactant hydrophobic tails was increased by about 20%. We may note here that structural predictions regarding pure SDS solutions from MD simulations based on standard *all-atom* force fields are also highly sensitive to the parameter sets modeling surfactant ionic head/Na^+^ interactions as well as the explicit water model used.^34,36^

Recent MD simulations of pure SDS and binary SDS/CAPB solutions^32^ without any counterion and any added salt show that truncated Lennard-Jones and coulombic potentials used in conjunction with the most recent MARTINI force field version 3.0b yield surfactant assemblies that overall agree with available *all-atom* simulation and experimental data.^52^ These simulations were performed on relatively short time scales (2 μs) and they do not discuss salt effects on micelle morphologies.

In the present study, we investigate the effect of alkali/halide salts (such as LiCl or NaCl) up to a concentration of *c*_salt_ = 2.5 M on binary 3:1 SDS/CAPB solutions from MD simulations based on a MARTINI-like CG approach. Besides assessing the ability of MARTINI simulations to provide meaningful data regarding mixed surfactant salty aqueous solutions (which are much less studied using *all-atom* and CG simulations compared to monocomponent solutions), we may again note that CAPB (up to a fraction of 0.3) already and largely enlarges micelle sizes in SDS/CAPB solutions, whereas it has a weak effect on other specific micelle properties (sphere to rod critical concentrations and hydrodynamic diameters).^10^ To complement experiments, exhaustive simulations of SDS/CAPB 3:1 solutions will allow one to investigate the global and local compositions of their mixed micelles, for instance. These will provide new data to understand the main features of these kinds of widely used binary solutions.

The composition of our SDS/CAPB surfactant solutions corresponds to standard conditions in the personal care/cosmetic industry (i.e., the total surfactant concentration is about 400 mM). We mostly use the recent MARTINI v3.0b force field parameter set, even if we refine the parameters regarding hydrophobic surfactant bead/water interactions to match micelle size experimental data with no added salt. Importantly, we simulate our systems without truncating the coulombic potential, by means of an efficient fast multipole moment, FMM, scheme recently proposed by one of us.^53^ However, we will briefly discuss here also the artifacts arising from truncating long-range Coulombic effects from simulations of SDS/CAPB solutions with no added salt. We also made an attempt to investigate the relation between the strength of salt cation/SDS anionic head interactions and micellar sizes and morphologies by considering different sets of CG force field parameters to specifically handle these interactions.

To the best of our knowledge, all MARTINI studies dealing about surfactant solutions rely on MD simulations performed at most at the 10 μs scale. However, as shown by Shaw and collaborators in the case of the folding process of a small protein in the aqueous phase,^54^ MD simulations at the 10 μs scale are still too short to warrant an exhaustive sampling of potential energy surface, PES, of complex molecular systems. To circumvent potential drawbacks arising from (relatively) too short MD simulations, we investigate our surfactant solutions using a Hamiltonian replica exchange (HRE) approach (see ref 55 and the references cited therein) combined with MD simulations at the 2–3 μs scale. Among the numerical approaches to enhance the sampling of the molecular system PES (see among others ref 56 for micellar solutions), the HRE scheme is a powerful technique to speed up PES investigations. For instance, in the study of Ostermeir and Zacharias,^55^ the folded geometry of a small peptide is reached within 20 ns using HRE simulations and still not observed along a single MD run at the 60 ns scale (see also the studies cited in ref 57).

Besides investigating salt effects on micelle sizes, morphologies, and viscosities, we also discuss a popular theoretical framework to understand micelle thermodynamics, a framework that relies on the concept of surfactant packing parameters in micelles and on surfactant pseudo-(micro)­phase distributions.^58,59^ A packing parameter *P* value is the ratio between the surfactant molecular mean volume *v̅*_chain_ and the product of the mean surfactant length *l̅*_chain_ times the mean surface area *a̅*_chain_ of the surfactant ending moiety at the micelle surface. For surfactant chains within spheroidal and cylindrical sections of a micelle, ideal *P* values are 1/3 and 1/2. For a surfactant chain located in a micelle concave section (of a branched wormlike micelle, for instance), its *P* value is about 0.8. The pseudophase framework states that an equilibrium exists in a micellar solution between the different kinds of surfactant chains, defined from their *P* value.^60^ The ability of this kind of approach to model the salt response of pure SLES_1_ solutions in the presence of small cosolutes (namely, fragrance molecules that are treated as a weak perturbation of pure surfactant solutions) has already been discussed^23^ contrary to mixed anionic/zwitterionic micellar aggregates. Anticipating our results, we will show here that a pseudophase approach is able to model this kind of mixed solutions by only taking into account the anionic surfactant (when it is, *a priori*, the most abundant species).

The MARTINI-like CG approach relies on a simple electrostatic picture of anionic/zwitterionic species. Only a very few static point charges modeling their charged moieties (such as SDS anionic heads) are taken into account. Despite more sophisticated CG approaches, including dynamical polarization effects, having been proposed to model pure SDS solutions,^61^ we have to keep in mind that there are still discrepancies between experiments and even the most sophisticated *all-atom* polarizable force fields (with parameters only set to reproduce high-level quantum chemistry data) to study the hydration of single ions (see ref 62 and the studies mentioned therein). This guides us to discuss how to reasonably take into account CG simulation findings, i.e., are they expected to predict mixed anionic/zwitterionic micellar solution data at a high level of accuracy for a specific added salt (such as NaCl) or do they provide reliable trends of solution properties (including their dependence on the amount of added salt) in order to build efficient and accurate thermodynamical models devoted to micellar solutions, for instance. Note that for readability purposes, we provide the full list of abbreviations used below in the Supporting Information.

## 2 Computational Details

### 2.1 Molecular Dynamics and Hamiltonian Replica Exchange Simulations

We perform NPT MD simulations using a Nosé–Hoover barostat^63^ (the coupling constant is set to 2.4 ps), whereas we use a Langevin thermostat approach^64^ to perform NVT runs. The target temperature is 300 K. The equations of motion are solved using a standard Multiple Time Steps (MTS) algorithm.^63^ Three time steps are used: 2, 20, and 40 fs for intramolecular stretching/bending forces and Lennard-Jones/Coulombic short and long-range forces, respectively. The cutoff distance to compute short-range forces is set to 8 Å. With the above time steps, we do not observe any ‘crash’ along all our MD simulations (as defined in ref 65, which are inferred to arise from the combined used of *tiny* and *regular* beads). MD trajectories are sampled every 200 ps for analysis purposes. We perform HRE simulations using the protocol detailed in ref 55. Potential exchanges among replica are checked for each 2.4 ps. The potential energy of each replica is shifted by an amount of energy δ*U* ^HRE^ computed on the fly to reach an exchange-averaged rate among replica of 40%. *δU* ^HRE^ is re-evaluated periodically by checking the exchange rate along 768 ps MD trajectory segments. Only the Lennard-Jones and Coulombic energy terms are scaled by the HRE parameter λ^HRE^ (which ranges from 0.3 to 1.0). We systematically consider 16 replica, and the MD runs were performed up to the 3 μs scale. The set of λ^HRE^ values that we used is provided in the Supporting Information. All simulations were performed using our own code POLARIS­(MD).^66^ The way we compute the long-range coulombic forces is detailed below in Section 2.3.

### 2.2 Simulated Systems

We consider for the present study up to six neutral SDS/CAPB 3:1 salty aqueous solutions corresponding to salt concentrations *c*_salt_ spanning from 0 to 2.5 M. For *c*_salt_ = 0 M, the surfactants represent 10% in mass of the solution (and a total surfactant concentration of *c*_surfactant_ = 400 mM). The solutions are modeled as cubic boxes, whose compositions are summarized in Table 1.

**Table 1:** Salt Composition of the Simulated Solutions^a^

*c*_salt_ (M)	*X*	*N*(NaCl)
0.0	0	0
0.2	0.5	346
0.6	1.7	1108
1.0	2.7	1773
1.3	3.3	2160
1.7	4.1	2646
2.5	5.4	3510

a Data correspond to the number *N* of NaCl added to the solutions. The amounts of SDS and CAPB chains are 482 and 165, respectively. X is the ratio between *c*_salt_ and *c*_surfactant_.

Water beads, SDS/CAPB chains, and salt ions (if any) are first randomly located at the nodes of a large enough cubic grid. The box dimensions are then relaxed by performing a preliminary NPT simulation. Box volumes are all converged within less than 10 ns. The NPT simulation final snapshots are optimized using a conjugate gradient algorithm until reaching a mean gradient norm per bead smaller than 10^–3^ kcal mol^–1^ Å^–1^. The optimized systems are the solution starting geometries for NVT production runs.

### 2.3 Coarse-Grained Force Field

In CG approaches, sets of atoms are taken into account as single beads. For SDS and CAPB, we provide our bead definition in Section S1 of the Supporting Information. This definition is equivalent to that used in earlier studies.^32,52^ Most bead sizes correspond to *regular* in MARTINI labeling,^49^ with the exception of salt ions (they correspond to *tiny* beads) and the CAPB carboxylate head groups (modeled as *small* beads). Water is modeled using *regular* MARTINI water beads W (each of them modeling four real water molecules).^49^ We do not make use of *large* (antifreeze) beads to model water as proposed in ref 67, as there is no liquid-to-solid transition along our simulations.

Interactions among bonded beads of SDS and CAPB chains are modeled using only harmonic stretching *U* ^stretch^ and bending *U* ^bend^ potentials. The stretching reference distance and harmonic constant are systematically set to 4.7 Å and 150 kcal mol^–1^ Å^–2^, respectively. The bending potential obeys

1 $$U^{\mathrm{bend}}=\frac{1}{2}\sum_{\mathrm{bead}\;\mathrm{triplets}}k_{\theta }{\left(\theta -\theta_{0}\right)}^{2}$$

The reference angle θ_0_ and constant *k*_θ_ are set to 180° and 5 kcal mol^–1^ rad^–2^, respectively, regardless of the triplet bead definition. Interactions among all nonbonded beads are modeled using a truncated Lennard-Jones *U* ^LJ^ potential

2 $$U^{LJ}=4\sum_{a}\sum_{b}^{*}\epsilon_{\mathrm{ab}}\left[{\left(\frac{\sigma_{\mathrm{ab}}}{r_{\mathrm{ab}}}\right)}^{12}-2{\left(\frac{\sigma_{\mathrm{ab}}}{r_{\mathrm{ab}}}\right)}^{6}\right]P\left(r_{\mathrm{ab}}\right)$$

where the superscript * means that the sum runs on unbounded pairs of beads and *P* is a five-order B-spline polynome allowing us to smoothly downscale to zero the magnitude of *U* ^LJ^ between 10.5 and 11.0 Å. We summarize the *U* ^LJ^ parameters in Table S1. For most bead pairs, they correspond to standard MARTINI v3.0b parameters.^49^

Aside from *U* ^LJ^, interactions among charged beads are modeled using a nontruncated coulombic *U* ^qq^ potential with a relative permittivity set to 30, as recommended for MARTINI approaches. The absolute charge of monoatomic anions/cations, SDS anionic heads, and CAPB betaine anionic/cationic moieties is set to +1*e*. Coulombic interactions are computed using the recent fast multipole moment, FMM, scheme proposed by one of us.^53^ The dimension of the smallest cubic FMM cells is about 9.0 ± 0.2 Å, and we consider only the first set of 26 periodic images of the main simulation box to model a bulk solution. This is shown to provide a reliable description of salty aqueous bulk solutions.^53^

As far as we know, only a few simulation studies were devoted to pure NaCl-like salty aqueous solutions using MARTINI force fields.^65,68^ We thus perform a preliminary study regarding such solutions to assess/refine the original MARTINI version 3.0b parameter set. The simulations were performed using our FMM approach to account for long-range interionic Coulombic interactions. Using close values to those proposed in the original MARTINI v3.0b force field, we have been able to reproduce a high dissociation degree for our monoatomic ion salt in neat water. From the parameter set (σ_ab_, ϵ_ab_) = (3.95 Å, 2.19 kcal mol^–1^), the salt dissociation degree linearly decreases from 95 to 83% as the salt concentration increases from 0.2 up to 2.0 M (see Figure S1). These dissociation degrees agree with the experiment^69,70^ and they are in line with available *all-atom* force field-based MD data for alkali/chloride salts (from LiCl to CsCl, see among others refs 71–75 ).

Regarding salt cation/SDS anionic head interactions, we first perform a series of potential of mean force (PMF) computations from MD NVT simulations at the 50 ns scale of a single salt cation/SDS pair dissolved in a 9000 water bead cubic box, using different LJ parameters ϵ_ab_ to handle the salt cation/SDS anionic head interaction (whereas σ_ab_ is systematically set to 3.95 Å). We compute four PMF profiles for ϵ_ab_ values that range between 0.48 and up to 3.48 kcal mol^–1^. These PMFs are shown in Figure 1: they all present a first minimum at about 4.3 ± 0.1 Å whose depth ranges from 0.4 to 3.1 kcal mol^–1^ for the force fields denoted as **R1** and **R4**, which correspond to larger and larger ϵ_ab_ values. These PMF first minimum depth values are in line with their counter parts from *all*-*atom* simulations for the cation series Li^+^ to Cs^+^ (they range from 0.7 to 2.6 kcal mol^–1^).^76^

**Figure 1:**
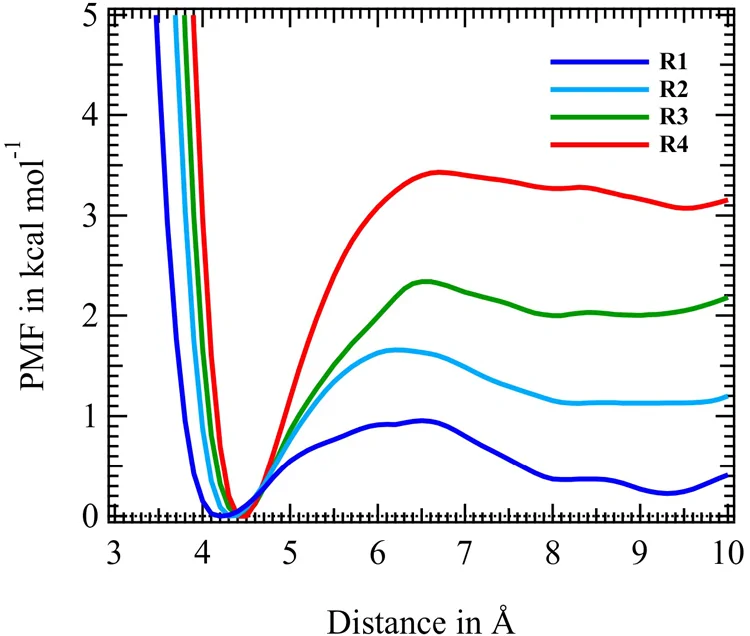
Salt cation/SDS PMFs in the aqueous phase. All PMFs are shifted so that their first minimum is set to zero. The force fields **R1**–**R4** correspond to the following LJ parameter sets to model salt cation/SDS anionic head interactions: (3.95 Å, 0.48 kcal mol^–1^), (3.95 Å, 1.28 kcal mol^–1^), (3.95 Å, 1.98 kcal mol^–1^), and (3.95 Å, 3.48 kcal mol^–1^), respectively. For PMF computation details, see Section S3 of the Supporting Information.

*A priori*, the nature of a specific monatomic salt (LiCl or NaCl, for instance) can be taken into account using appropriate sets of (σ_ab_, ϵ_ab_) parameters. However, because of the low amount of available experimental and simulation data, it is not obvious to assign our parameter sets **R1**–**R4** to a specific alkali/chloride salt. From our simulations discussed below, the parameter set denoted as **R3** allows us to reproduce micellar solution properties that overall agree with the experiment for added NaCl salt, whereas salts such as LiCl and KCl (or CsCl) correspond to intermediate **R1**/**R2** and **R3**/**R4** parameter sets, respectively.

We first performed a preliminary series of HRE simulations (using the FMM approach) regarding our 3:1 SDS/CAPB solution with no added salt to assess/refine the original MARTINI v3.0b force field in order to reproduce the expected magnitude of micelle sizes reported from the experiment, about 60 ± 10 for pure SDS solutions whose concentrations span from 50 to 170 mM.^11,13,15,77,78^ The original MARTINI force field v3.0b yields too small micelles compared to the experiment (their mean size is about 37). Modulation of the LJ parameters handling the interactions between surfactant hydrophobic beads and water allows us to readily modify micelle sizes: using (σ_ab_,ϵ_ab_) = (4.70 Å, 0.25 kcal mol^–1^), we were then able to reproduce micelle sizes that agree with the lower bound of the above experimental estimates. The latter refined ϵ_ab_ parameter is twice as small as the original one in the MARTINI force field v3.0b. See also Section 3.1 below for more details.

### 2.4 Clustering Analysis

We identify SDS/CAPB micelles along our MD trajectories using a simple clustering analysis scheme. At every simulation sampled snapshot, we define a micelle as a set of SDS and CAPB chains for which at least one distance between their hydrophobic beads is shorter than the reference distance *d*_micelle_ = 6 Å. From visual inspection of our MD trajectories, this basic rule prevents us from identifying a pathological set of SDS/CAPB chains (for instance, two small micelles lying at short range from each other) as a ‘micelle’. See also Figure 2c,d, where examples of the final set of micelles identified accordingly along our HRE runs are shown. According to our clustering approach, only a very few SDS and CAPB chains are not incorporated in ‘micelles’ (i.e., surfactant aggregates that comprise more than 10 chains) along our final MD segments, even if they can form small aggregates (comprising no more than five chains).

**Figure 2:**
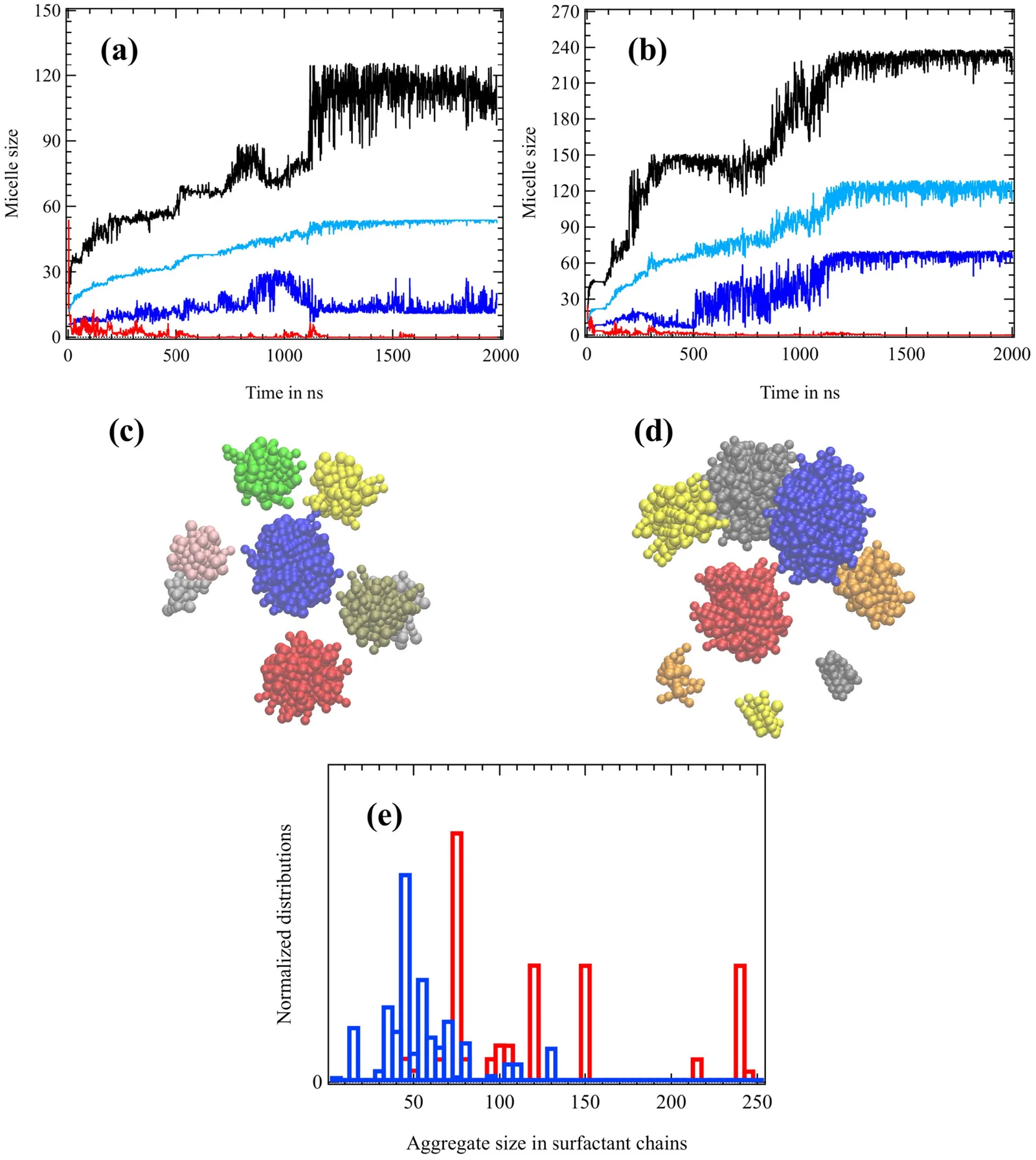
(a, b) Temporal evolutions of the min (dark blue), max (black) and mean (light blue) micelle sizes along reference HRE simulations taking into account or truncating long-range electrostatic interactions, respectively. The evolutions of the number of isolated surfactants (i.e., surfactants included within aggregates whose size is smaller than 5) are shown in red. (c, d) Snapshots from the later simulations. Counterions and water beads are not shown. The largest micelles are shown in dark blue. Because of the use of periodic conditions, small aggregates that we may observed in (c) and (d) snapshots belong to the main micelles with the same color. For (c) (simulations taking into account long-range interactions), three small micelles have been removed for readability purposes. (e) Micelle size-normalized distributions along the final MD segment for truncated (red) and untruncated (blue) coulombic potentials.

### 2.5 Shear Viscosities

We estimate the shear viscosity η̅_0_ of a micellar solution from the time integral η­(*t*) of the autocorrelation function of the pressure tensor off diagonal data Π_αβ_(*t*). The final η­(*t*) values are averaged according to the protocol of ref 79 from 64 independent simulations at the 1 ns scale in the NVE ensemble (once a thermalization step of 1 ns in the NVT ensemble using the Langevin thermostat is achieved). The MD simulations are performed using only two time steps (2 fs for intramolecular stretching and bending energy terms and 10 fs for all LJ and coulombic nonbonded interactions) and the FMM scheme detailed above. For each simulation *i*, pressure tensor data yield three viscosity estimates η_αβ_^*i*^(*t*) from the Green–Kubo relation. η_αβ_^*i*^(*t*) data from all independent simulations are then averaged to provide η­(*t*). The shear viscosity estimates, η̅_0_, discussed in the present study correspond to the mean values at the plateau region of η­(*t*) (averaged up to time *t* for which the standard deviation among η_ab_^*i*^(*t*) data is at most 40% of the final η̅_0_ magnitude^79^). For comparison purposes, we accordingly computed shear viscosity data η̅_0_ for pure alkali/halide salty aqueous solutions corresponding to salt concentrations of 0.0, 0.2, 0.6, 1.0, and 2.0 M. We systematically checked that the mean temperature at the end of the NVE runs matches the target one (300 K) within ±1 K.

## 3 Results and Discussion

We mostly discuss below data that correspond to the force field **R3**, for which the depth of the first minimum of the salt cation/SDS anionic head PMF amounts to 2.1 kcal mol^–1^. We only discuss data from the other force fields to place in perspective key **R3** results.

We denote below as the final MD segment the final longest segment of the reference HRE MD trajectory (which corresponds to λ_HRE_ = 1.0) along which the mean micelle size reaches a stable value. These segments are at least at the 1 μs scale. We assume below that the standard deviation to a mean value is an upper bound measure of the uncertainty tied to this average.

To discuss micellar morphologies (their level of sphericity), we compute the eccentricity *e* of a micelle from its moments of inertia *I* ^m^ according to

3 $$e=1-\frac{3I^{1}}{I^{1}+I^{2}+I^{3}}$$

The moments *I^m^* are sorted per increasing value from the smallest (*m* = 1) to the largest (*m* = 3). The micelle main inertia axes are denoted **Z** ^*m*^. The axis **Z** ^1^ is thus the longest micelle axis.

### 3.1 Original vs Our Revised MARTINI Approach

By means of our HRE/MD simulation protocol, we first investigate our surfactant solutions with no added salt to assess the effect of our revised force field parameter set and accounting for long-range electrostatics on SDS/CAPB aggregation only in the presence of counterions (i.e., the cations added to neutralize the solution total charge). To this end, we perform two series of HRE runs. The first series (denoted as SR) relies on truncating coulombic interactions for interbead distances larger than 11 Å and on the original MARTINI force field parameters v3.0b. The second series (denoted LR) corresponds to our revised force field with accounting for long-range electrostatics.

The SR and LR temporal evolution of the smallest, largest, and mean sizes of micelles that are observed along the HRE reference simulations is shown in Figure 2a,b. Regarding SR, the mean micelle size increases by steps until reaching a large and stable value of 122 ± 12 after 1.1 μs. The smallest and largest micelle mean sizes are then 66 ± 8 and 231 ± 12, respectively. Only four main micelles dominate in the solution; see the micelle size distribution shown in Figure 2e, and the solution is a mixture of spheroidal and elongated micelles (their mean eccentricities *e̅* are smaller and larger than 0.15, respectively, see Figure S3). All these data are in line with those reported in ref 32 for 2:1 SDS/CAPB solutions at the 400 mM scale from a single MD simulation at the 2 μs scale and performed using the original MARTINI v3.0b force field (with truncated coulombic interactions).

Long-range electrostatics strongly disfavor large micelle size as shown by LR data. Even by using our revised force field parameter set (which yields about twice as large micelles compared to the original MARTINI force field v3.0b; see Section 2.3), the final mean and largest micelle sizes are only 53 ± 1 and 112 ± 13, respectively. The LR mean micelle size distribution is quasi-Gaussian and centered at about 50 (see Figure 2e), and most LR micelles are spheroidal. However, the mean eccentricity values *e̅* of about 7% of LR micelles are larger than 0.15, and we may even note a micelle whose size is 107 with *e̅* > 0.3. Hence, the spheroidal-to-elongated SE (also called “sphere-to-rod”) transition is also already achieved in our solution with no added salt when taking into account long-range electrostatic effects. Experimentally, the SE transition of a SDS/CAPB 3:1 solution with no added salt is reported to occur at about 200 mM (and at about 150 mM for a 2:1 solution)^10^ and the micelle size overall slowly increases as the surfactant concentration increases (as inferred from the experimental hydrodynamic radius data of ref 10). We may thus argue that our LR simulations are in better agreement with experiments dealing about SDS/CAPB 3:1 solutions than SR ones.

Only a few single surfactant chains (mainly SDS) not incorporated in any micelle are observed along both the SR and LR final MD segments. Micelle compositions in SDS/CAPB chains are in line with the global solution composition (they are in 3:1 ratio), on average, and regardless of the micelle size. However, the proportions of SDS and CAPB chains in large micelles (size >75) can vary at most by 5% compared to the global composition, whereas they can vary up to 10% in smaller micelles.

Long-range effects are thus a key factor modulating the size of mixed SDS/CAPB micelles as already shown for pure SDS solutions.^50–52^ When accounting for them, our refined CG force field predicts a mean size for SDS/CAPB micelles that corresponds to the lower bound of experimental data for pure SDS solutions (with no added salt), which mostly span from 53 to 75 for surfactant concentrations at the 50 mM,^13,15,77,78^ 100 mM,^80,81^ and 170 mM^11^ scales. Nevertheless, larger mean micelle sizes (up to twice as large) are also experimentally reported for pure SDS solutions at the 170–300 mM scale^8–10,12^ and the mean sizes of mixed SDS/CAPB micelles (with no added salt) are experimentally reported to be larger than for SDS or CAPB solutions by about 30%,^10^ regardless of the solution composition. Note again here that the reported experimental micelle sizes vary up to a factor of 2 for a specific solution (see data from refs 11 and 12).

Another striking difference arising from accounting for or omitting long-range electrostatics arises from the micelle degree of neutralization by counterions (see Section 3.4 below for more details). Accounting for long-range effects yields a mean neutralization degree that is 53 ± 7%, whereas this degree is only 29 ± 5% using the original truncated MARTINI approach. The LR degree of neutralization matches most experimental data for pure SDS solutions (with no added salt), which are included between 50 and 60% for 50 to 500 mM solutions.^9,81–83^ Note, however, that neutralization degrees amounting to 70–80% for 50 mM SDS solutions have also been reported.^8,12,80^ Anticipating our data with added salts (which show micelle sizes increasing as the neutralization degree increases), the latter result shows that there is a complex interplay between micelle sizes, the degree of neutralization, and long-range electrostatics in micellar solutions.

### 3.2 Salt Effects on Micellar Growth

For added salt concentrations of up to 2.5 M, micelle size data along final MD segments are summarized in Table 2 and the averaged largest and mean micelle sizes are plotted as a function of the ratio *X* in Figure 3. Temporal evolutions of mean, smallest, and largest micelle sizes for each salt concentration are reported in the latter figure (*c*_salt_ = 1 M) and in Figure S2.

**Figure 3:**
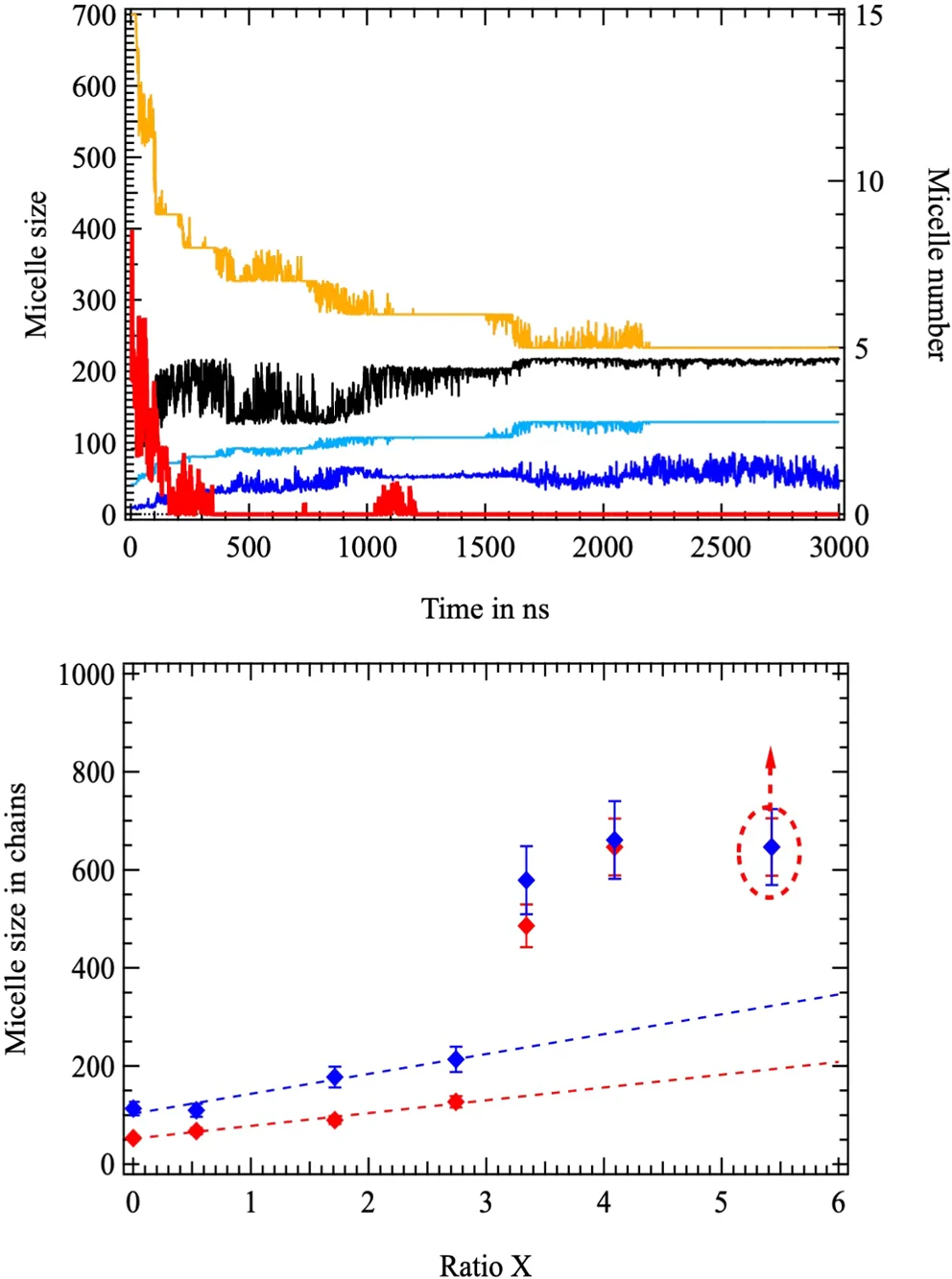
Top: temporal evolutions of the smallest (dark blue), mean (light blue), and largest (black) micelle sizes along the final MD segment for *c*_salt_ = 1 M. Orange line (right axis): temporal evolution of the micelle numbers. Red line: temporal evolution of the numbers of isolated chains or of surfactant assemblies comprising no more than five chains. Down: largest (blue markers) and mean (red markers) micelle sizes as a function of the ratio *X*. Dashed lines: best linear fit for data corresponding to *X* < 3 (Pearson’s regression coefficient is larger than 0.98 for both kinds of data). Circled data and the arrow mean that the largest and mean sizes are larger because of the interaction of the main ‘infinite’ micelle with two of its own periodic images for *X* = 5.4 (*c*_salt_ = 2.5 M).

**Table 2:** Averaged Micelle Dimensions^a^

*c*_salt_ (M)	*X*	min	mean	max	*L̅*_m_
0.0	0.0	14.1	53.5	111.5	22.4
0.2	0.5	31.7	64.3	**101.1**	24.3
0.6	1.7	43.5	90.0	168.0	26.1
1.0	2.7	53.8	126.3	213.8	29.7
1.3	3.3	508.1	554.8	**608.9**	34.4
1.7	4.1	557	587	**608**	115
2.5	5.4		∞		na

a Min, mean, and max: smallest, mean, and largest micelle sizes (expressed in surfactant chains) over final MD segments, respectively. For *c*_salt_ = 2.5 M, ∞ means that a single micelle is observed, a micelle that extends outside the replicated simulation box and interacts at both its ends with two of its own periodic images; see Figure 4c. *L̅*_m_: mean longest micelle length in Å, measured from SDS anionic and CAPB carboxylate beads along the inertia axis **Z** ^**1**^.

The mean, smallest, and largest micelle sizes linearly increases up to *c*_salt_ = 1 M, which is in line with the experiment for pure SDS aqueous solutions at the 50/170 mM scale with added LiCl and NaCl salts.^11,13,77^ Then, an abrupt transition toward much larger micelles starts for *c*_salt_ included between 1.0 and 1.3 M, with the appearance of ‘giant’ micelles comprising all the surfactant chains at *c*_salt_ = 1.7 M. This transition phase ends at *c*_salt_ = 2.5 M with the formation of single ‘infinite’ wormlike micelles that include all the surfactants chains and extend outside the replicated simulation box by interacting with two of their own periodic images at both their ends (two different ‘infinite’ wormlike micelles are observed for *c*_salt_ = 2.5 M).

The appearance of long wormlike micelles is tied to the shear viscosity peak in salt curves, a peak that we may reasonably infer to be located within *X* = 3 and 5 for pure SDS and mixed SDS/CAPB solutions with added NaCl salt, respectively (see our discussion in the Introduction). The latter peak estimate agrees with our CG simulations based on the force field **R3**, which predict the appearance of ‘infinite’ wormlike micelles at *c*_salt_ = 2.5 M, i.e., for *X* = 5.4.

### 3.3 Micelle Morphologies and Compositions

Along final MD segments, the mean SDS and CAPB molecular lengths *l̅* are 13.97 ± 0.03 and 23.0 ± 0.1 Å regardless of the amount of added salt, respectively. Accounting for the implicit ending oxygen and hydrogen atoms for both kinds of chains (i.e., for their van der Waals radii and for their chemical bond lengths to the surfactant ending sulfur, carboxylate, and methyl carbon), *l̅* is estimated to be about 15.8 (SDS) and 24.7 (CAPB) Å. These lengths are smaller by about 1 Å compared to the ones computed according to the relation proposed by Tanford^60^ to investigate micelle growth thermodynamics: 16.7 (SDS) and 25.5 (CAPB) Å. We systematically use in the present study the standard MARTINI ‘bonding’ distance set to 4.7 Å to model all intrachain ‘stretching’ interactions. Tanford’s chain lengths can be thus, a priori, reproduced just by slightly increasing the latter distance by about a few pm.

Because of the longer length of CAPB chains compared to SDS, protruding CAPB ending carboxylate beads lie outside the micelle surface defined by SDS anionic and CAPB ammonium beads by about 6.6 ± 1.2 Å, regardless of the micelle size and morphology (see Figure 4). This supports the common assumption regarding the surface of such mixed anionic/zwitterionic assemblies.^84^

**Figure 4:**
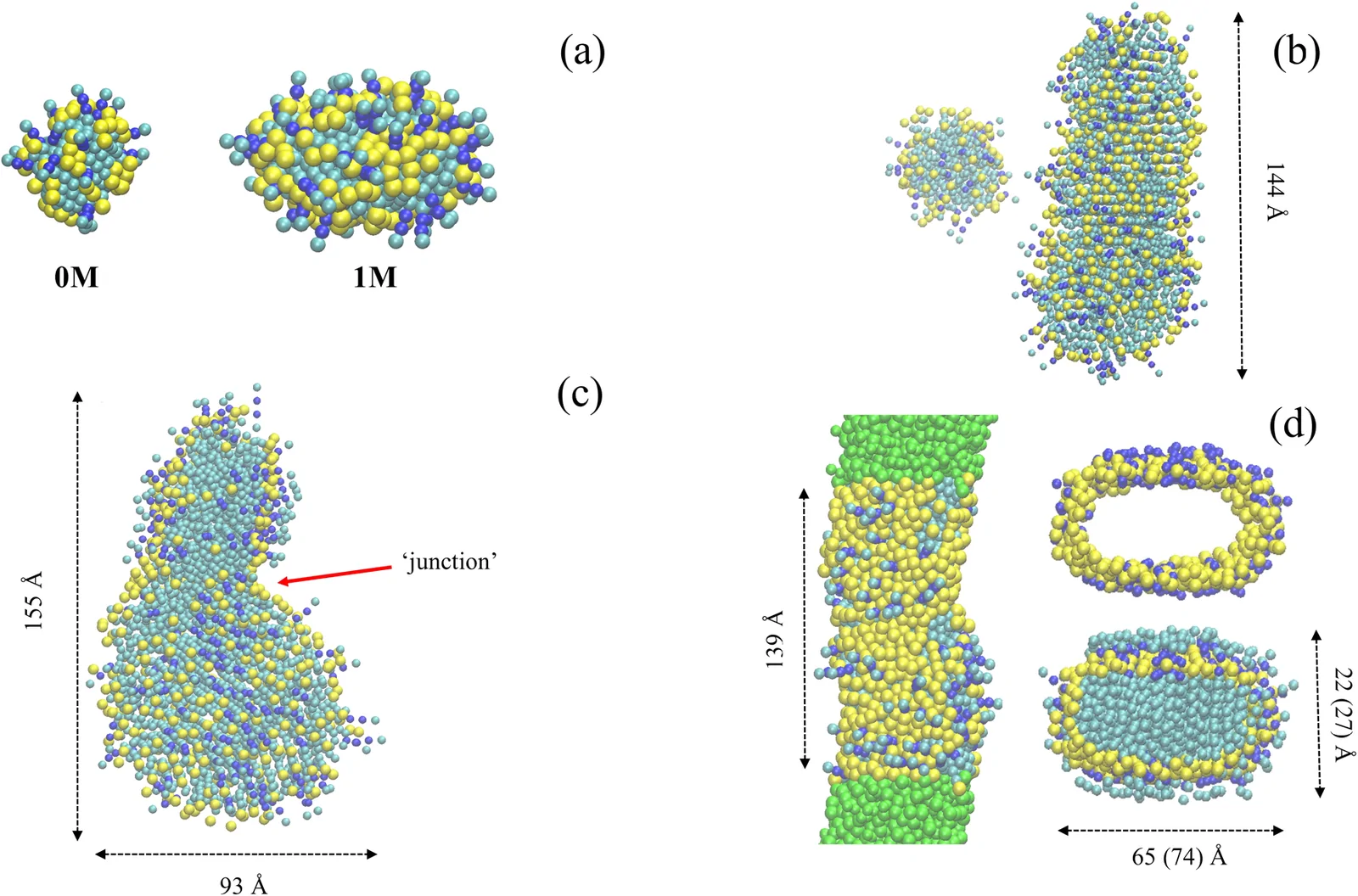
Examples of micelles along final MD segments for *c*_salt_ = 0.0 and 1.0 (a), 1.3 (b), 1.7 (c), and 2.5 M (d). SDS anionic heads are shown in yellow, CAPB amide and ammonium moieties are shown in dark blue, and alkyl and carboxylate beads are shown in light blue. For *c*_salt_ = 1.7 M (c), the red arrow shows a local ‘junction’-like concave section of a ‘giant’ micelle. For *c*_salt_ = 2.5 M (d), the two periodic images of the main wormlike micelle are shown in green. On the right, we show sections orthogonal to the main micelle axis (the distances are measured from SDS anionic and CAPB carboxylate beads, and the latter ones are provided in parentheses).

Aside from micelle eccentricity data, we also investigate micelle morphologies by computing the three mean distances *R̅*_bead_^*m*^ for a specific surfactant type of beads. These distances are measured from the micelle COM and averaged among beads whose angle between their corresponding vector **R**_bead_^*m*^ and the *m* ^*th*^ inertia axis **Z** ^*m*^ is smaller than Θ^*m*^ = 3*πI* ^*m*^/4­(*I* ^1^ + *I* ^2^ + *I* ^3^); see Figure 6a. The resulting mean distances are then further averaged over all micelles of equal size identified along a final MD segment.

#### 3.3.1 Low Added Salt Concentrations

We plot in Figure 5 the mean eccentricity value *e̅* for micelles of equal size along the final MD segment as a function of their size for *c*_salt_ = 1 M. This plot shows a mixture of spheroidal (*e̅* ≤ 0.15) and elongated micelles (*e̅* > 0.15) to coexist in the solution. Micelles up to a size of 130 chains can be assumed to be overall spheroidal, whereas elongated micelles correspond to larger sizes. The ratios of the moments *I* ^2^ and *I* ^3^ for elongated micelles span from 1.10 to 1.30. The core of elongated micelles is more likely an oval tube than an ideal cylinder. These results hold for weaker added salt concentrations, with however micelles smaller in size and more and more spheroidal as *c*_salt_ decreases. For readability purposes, we will denote the ending and central sections of an elongated micelle along its longest axis **Z** ^1^ as ‘end-cap’ and ‘cylindrical’ sections, respectively.

**Figure 5:**
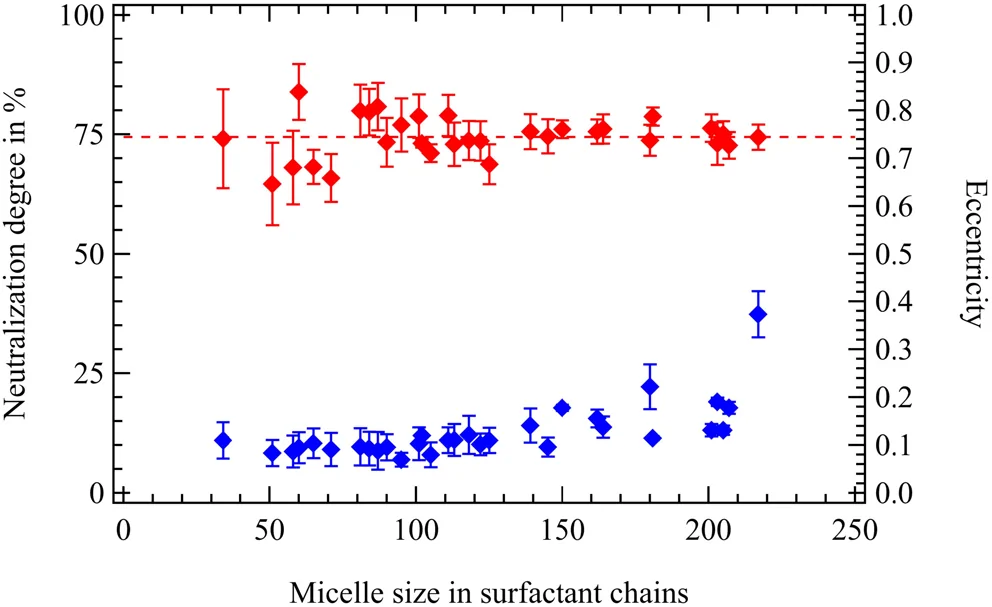
Micelle degree of neutralization (red markers, left axis) and mean values *e̅* of the micelle eccentricity (blue markers, right axis) as a function of the micelle size for *c*_salt_ = 1 M (*X* = 2.5) along the final MD segment. Dashed red line: neutralization degree averaged over all the micelles. The error bars correspond to standard deviations of the mean values.

With regards to SDS chains of elongated micelles for which the projection *Z*_anionic_^1^ of their anionic bead on the micelle main axis **Z** ^1^ obeys |*Z*_anionic_^1^| ≥ *R̅*_anionic_^1^ – *l̅*_SDS_ (condition **1**), we note that the absolute value of the projection *Z*_ending_^1^ of their ending hydrophobic bead differs from *R̅*_anionic_^1^ – *l̅*_SDS_ (in absolute value) by 0.9 ± 0.3 Å, on average, for *c*_salt_ values spanning up to 1 M. Hence, these specific SDS chains are spherically distributed, with their anionic and ending hydrophobic beads located at the surface and at the center of half of a sphere of radius *l̅*_SDS_ and centered at ± (*R̅*_anionic_^1^ – *l̅*_SDS_). Conversely, SDS chains that obey |*Z*_anionic_^1^ |< *R̅*_anionic_^1^ – *l̅*_SDS_ (condition **2**) are locally packed with their neighbors in nonoverlapping parallel conformations. See an example in Figure 4b. These results (which hold for CAPB chains) allow us to readily sort out end-cap and cylindrical SDS chains, which obey the latter conditions **1** and **2**, respectively.

#### 3.3.2 High Added Salt Concentrations

For added salt concentrations *c*_salt_ larger than 1 M, the micellar growth rate abruptly increases up to single ‘giant’ micelles (*c*_salt_ = 1.7 M) and then single ‘infinite’ wormlike micelles (*c*_salt_ = 2.5 M), which interact with two of their own periodic images at both their ends; see Figure 4. Note that very large micelles (including up to all surfactant chains) are already observed for *c*_salt_ = 1.3 M. Such an abrupt micellar growth rate change is in line with the experiment for pure SDS solutions at the 50 mM scale (on a weaker range of added salt amounts that yield, however, larger ratio *X* values).^13^

For the ‘infinite’ wormlike micelles (*c*_salt_ = 2.5 M), the mean ratio between their inertia moments *I* ^2^ and *I* ^3^ is about 1.10 ± 0.1. These micelles are thus an infinite oval tube with a bilayer-like core section made of a highly ordered parallel packing of nonoverlapping surfactant hydrophobic chains. There is no empty volume along the main micelle axis **Z** ^1^. Note, however, that a few (rare) snapshots along the final MD segment *c*_salt_ = 2.5 M exhibit a ‘giant’ disklike micelle (see below).

For *c*_salt_ = 1.7 M, an equilibrium exists between two ‘giant’ micelles including all surfactant chains, and both appear to arise from the merging of the disklike micelle with a spheroidal one; see one of these assemblies in Figure 4b,c. The chain geometry within the disklike micelle inner core is bilayer-like, whereas it corresponds to a ‘junction’-like packing (i.e., chains are buried in the local concave portion of the micelle surface) within the disklike/spheroidal merging domain. While disklike micelles are rarely mentioned for anionic/zwitterionic surfactant solutions, they have been reported to occur in 4:1 SDS/CAPB solution with a less added amount of lauryl acid^31^ and, interestingly, in pure SLES_2_ solutions at the 200 mM scale with added salt KCl near the solution viscosity peak by cryo-TEM imaging.^20^ The diameter of the SLES_2_ disklike micelles is at the 50–100 Å scale, in agreement with our SDS/CAPB simulations.

#### 3.3.3 Local Micelle Compositions

For each micelle (at the exception of the ‘infinite’ ones), we compute the amounts of chains *n̅*_SDS_^*m*^ and *n̅*_CAPB_^*m*^ in the conic domains defined by the micelle main inertia axes **Z** ^*m*^ and the angles Θ^*m*^; see Figure 6a. We then estimate the ratios *r̅*_surfactant_^*m*^ = *n̅*_CAPB_^*m*^/ *n̅*_SDS_^*m*^ normalized by the ratios of the total numbers of SDS and CAPB chains within the micelle. Ratios *r̅*_surfactant_^*m*^ are plotted as a function of the micelle size for *c*_salt_ = 1.3 M in Figure 6b. These ratios are about unity (within about 10%), on average, for small/spheroidal micelles up to 70 chains, which shows a homogeneous distribution of SDS and CAPB surfactants. For larger micelles, the ratios *r̅*_surfactant_^1,2^ strongly decrease as the micelle size increases and the ratio *r̅*_surfactant_^3^ exhibits the opposite trend. Compared to their global compositions, ‘end-cap’ and ‘cylindrical’ domains of large micelles are thus largely enriched in SDS and CAPB chains, respectively. These differences in local composition are observed in all solutions starting at *c*_salt_ ≥ 0.6 M (see Figure S4). They favor a reinforcement of salt cation/SDS anionic bead interactions within ‘end-cap’ domains.

**Figure 6:**
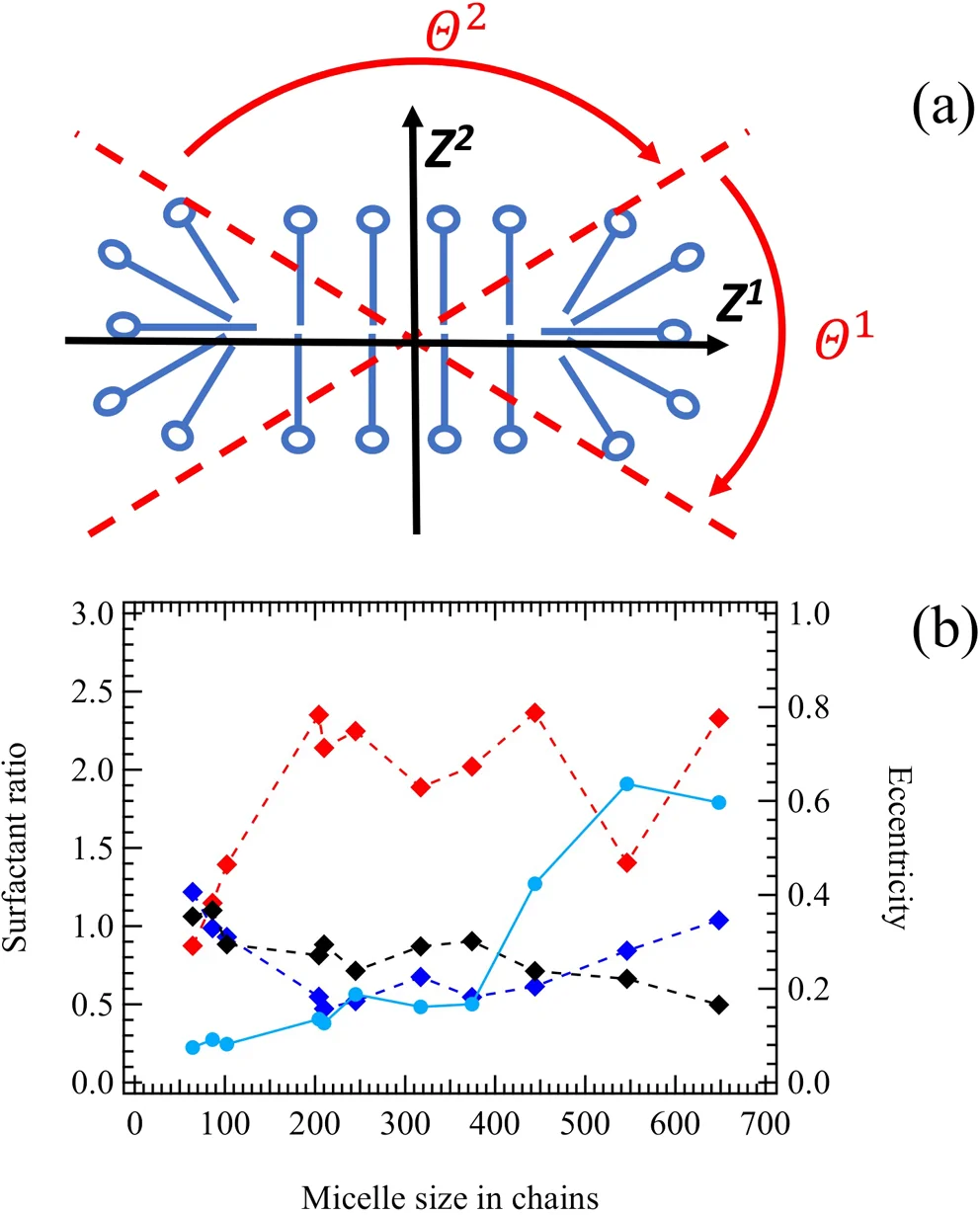
(a) Definition of the conic domains (corresponding to the angles Θ^1,2^ defined in the text) whose symmetry axes are the inertia axes **Z** ^1^ and **Z** ^2^ of a micelle (SDS and CAPB chains are both schematically represented in blue). The third conic domain corresponds to the axis **Z** ^3^ orthogonal to the latter axes. (b) Normalized ratios *r̅*_surfactant_^*m*^ as a function of the micelle size for *c*_salt_ = 1.3 M (X = 3.3) (left axis) and micelle eccentricities (light blue markers, right axis). Markers in black, blue, and red: ratios *r̅*_surfactant_^*m*^ within the conic domains corresponding to axes **Z** ^*m*^, with *m* = 1, 2, and 3, respectively. Only data corresponding to micelles whose occurrence is larger than 30 along the final MD segment are shown.

The chain composition is heterogeneous in the ‘infinite’ micelles, with large portions of the micelles made of only SDS chains and other portions enriched in CAPB; see Figure 4. As argued above, these local compositions favor large salt cation/SDS anionic head electrostatic interaction networks that stabilize the micelle surface.

#### 3.3.4 Strength of Salt Cation/SDS Anion Head Interactions

We have also investigated 3:1 SDS/CAPB solutions for an added salt concentration of 2.5 M (*X* = 5.4) using the force fields **R2** and **R1** for which the salt cation/SDS anionic head interactions are more and more weakened. Along final MD segments, we only observe ‘giant’ micelles comprising all surfactant chains. For **R1**, the morphology of 82% of these ‘giant’ micelles corresponds to an oval tube as for the ‘infinite’ micelle at *c*_salt_ = 2.5 M (see an example in Figure 7a), whereas the morphology of the remaining 18% corresponds to an oblate ellipsoid whose main axis is about 130 Å long. For **R2**, the morphology of all the ‘giant’ micelles is also an oblate ellipsoid equivalent to the force field **R1**. This suggests that the solution viscosity peak has still not been reached with force fields **R1**/**R2** at *c*_salt_ = 2.5 M, contrary to using force field **R3**.

**Figure 7:**
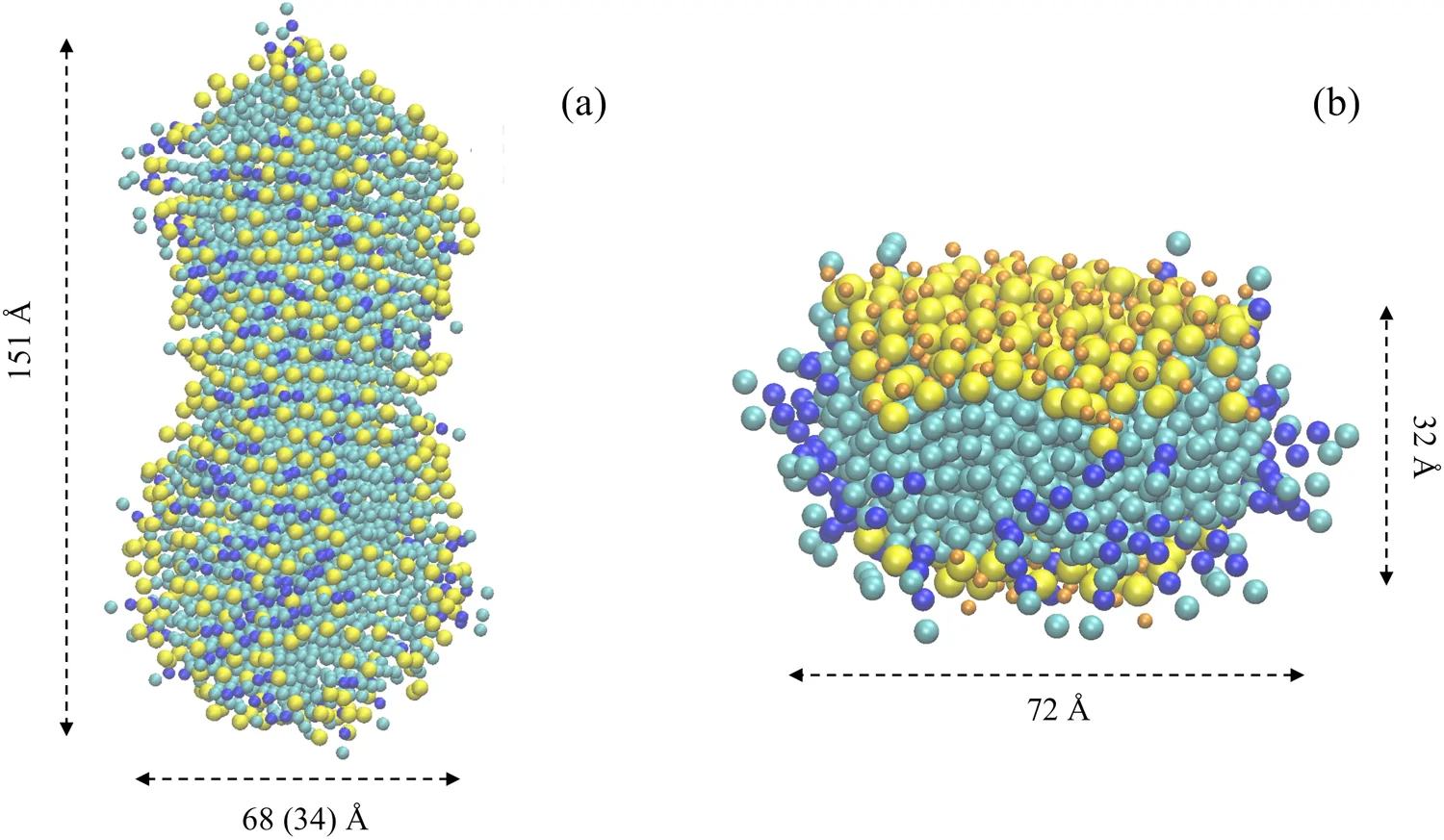
Examples of micelles along final MD segments using (a) force field **R1** with *c*_salt_ = 2.5 M and (b) force field **R4** with *c*_salt_ = 1.0 M. In the latter case, salt cations at the micelle surface are shown in orange. For (a), the distance in parentheses corresponds to the smallest micelle axis.

Similarly, we also simulate our surfactant solution with *c*_salt_ = 1 M using the force field **R4**, which corresponds to strong salt cation/SDS anionic head interactions. In this case, an equilibrium of only disklike micelles emerged with their size ranging from about 90 to 400 chains. Contrary to force field **R3**, SDS chains are then all concentrated within the bilayer-like micelle inner core, whereas CAPB chains are all located within the micelle rims. Close to the solution viscosity peak, cryo-TEM imaging^20^ shows the presence of disklike micelles within a mixture of mostly elongated/rod-like micelles for pure SLES_2_ solutions with added salt KCl. Even if this should be discussed further, we consider the force field **R4** as unphysical as it severely overestimates the strength of salt/SDS interactions (and this force field does not correspond to any kind of alkali/halide salts).

The above results show the sensitivity of SDS/CAPB aggregation phenomena to the strength of salt cation/SDS anionic head interactions. Moreover, we may argue that force fields **R3** and **R1**/**R2** model more likely the salts NaCl and LiCl, respectively.

### 3.4 Micelle Charge Neutralization

We plot in Figure 8a the mean radial distribution functions of salt cations centered at SDS anionic beads computed along final MD segments for all *c*_salt_ values and force field **R3**, as well as for force fields **R1** to **R4** and *c*_salt_ = 1 M. These functions present all a first sharpened peak located at *R*_max_^1^ = 4.3 ± 0.1 Å, a peak that extends up to *R*_min_^2^ = 6.3 ± 0.1 Å. This peak corresponds to salt cations neutralizing/screening the total micelle charge *Q*_m_. As the mean distance of this kind of cations to micelle COM’s is close to that of SDS anionic beads (within about 0.5 Å, regardless of the micelle size and morphology), we assume them to counterbalance the charge *Q*_m_. We denote below *p̅*_neutralization_, the mean degree of neutralization (in percent) of micelles from these cations.

**Figure 8:**
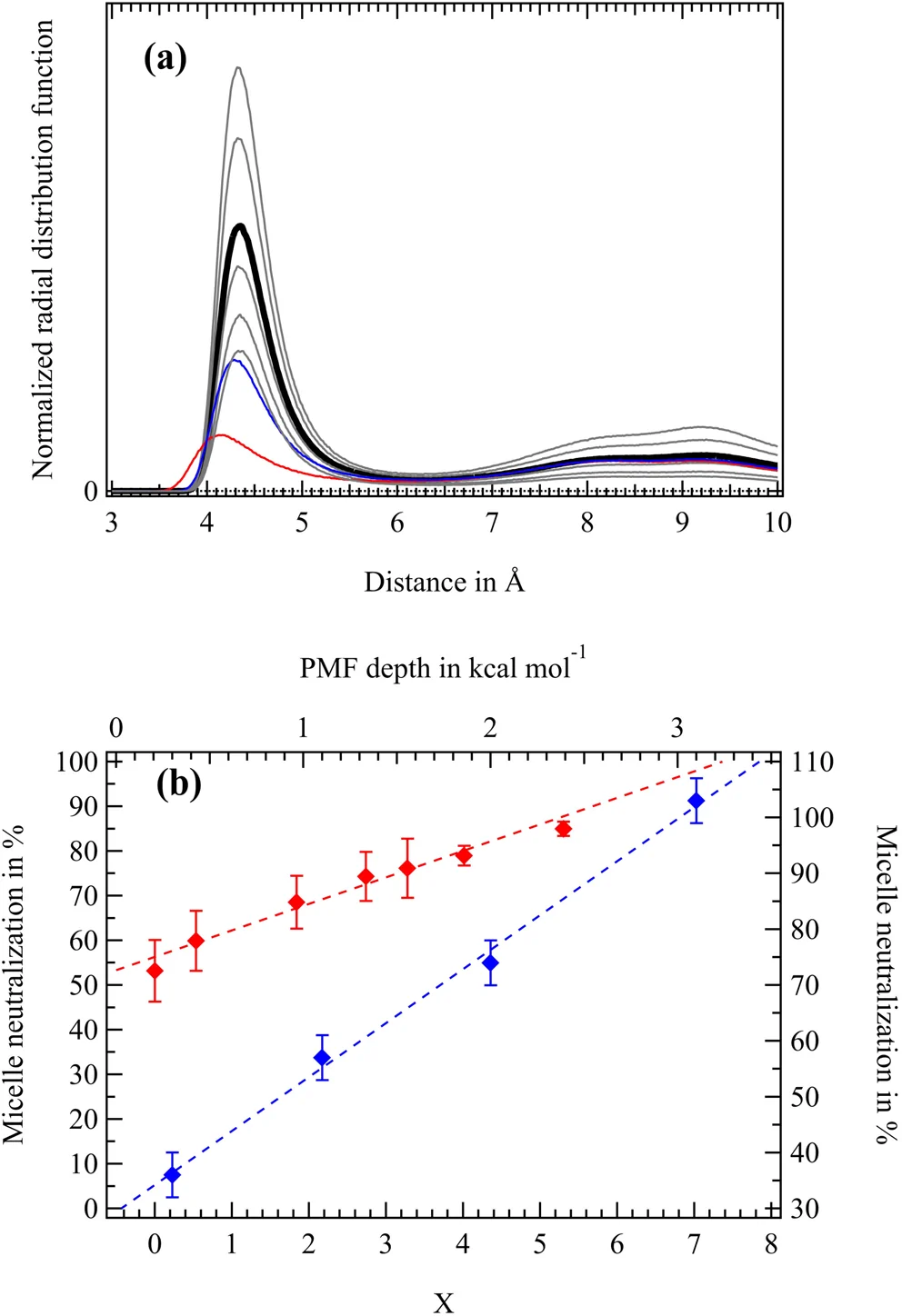
(a) SDS anionic bead/salt cation mean radial distribution functions computed along final MD segments. Black bold line: distribution for force field **R3** and *c*_salt_ = 1 M. Gray lines: distributions for force field **R3** and *c*_salt_ ≠ 1 M (the higher the peak, the larger the *c*_salt_, from 0.2 to 2.5 M). Red and blue lines: distributions for *c*_salt_ = 1 M and force fields **R1** and **R2**, respectively. (b) Mean degree of neutralization *p̅*_neutralization_ as a function of the ratio *X* (red markers, left and bottom axes) and as a function of the cation/SDS first PMF depth for force fields **R1** to **R4** (blue markers, right and upper axes, PMF data are discussed in Section 2.3). Dashed lines: best linear correlation fits (Pearson’s correlation coefficients are both larger than 0.97).

From the plots of Figure 8b, *p̅*_neutralization_ depends linearly on both the ratio X (for force field **R3**) and the force field for *c*_salt_ = 1 M. *p̅*_neutralization_ values span from 36 to 84%, a range of values that is in line with *all*-*atom* simulations for pure SDS solutions.^34,36^ Note that for force field **R4** and *c*_salt_ = 1 M, *p̅*_neutralization_ reaches a value of 103%.

For force field **R3**, we assume the equilibrium constant *K*_*d*_ corresponding to the dissociation of close-lying salt cations from micelle surfaces to obey

4 $$K_{d}=\frac{(c_{\mathrm{salt}}+c_{\mathrm{charged}})c_{\mathrm{charged}}\gamma^{*}}{1-c_{\mathrm{charged}}}$$

where *c*_charged_ is the amount of unneutralized SDS chains by salt cations and γ* is the product of the activity coefficients of bulk free salt cations and of non-neutralized SDS chains at micelle surfaces (according to an extension of the Debye–Hückel approach for ionic concentrations up to 2 M^85^ and using the parameters of ref 86). For force field **R3**, the *K*_*d*_ value linearly increases from 0.07 M (no added salt) up to a constant value of 0.27 ± 0.01 M for *c*_salt_ ≥ 1.3 M (see Figure S5). Our *K*_d_ data are overall in line with the mean value used in ref 23 (0.26 M) to model the effect of cosurfactants on SDS micellar solution viscosities in the presence of NaCl salt. Note that ignoring the ionic activity coefficient γ* yields about 50% larger *K*_d_ values.

With regards to a given *c*_salt_ concentration, correlation exists between micelle sizes/morphologies and *p̅*_neutralization_ values; see Figure 5 for *c*_salt_ = 1.0 M. The *p̅*_neutralization_ value for a specific micelle size matches the global one averaged over all micelles at a given *c*_salt_, within the error bar. For *c*_salt_ ≤ 1.3 M and micelle sizes larger than 130 chains, the local neutralization degrees in ‘end-cap’ micelle domains are larger by 5 up to 9% than in ‘cylindrical’ sections, which echoes the relatively larger amounts of SDS chains in ‘end-cap’ domains compared to ‘cylindrical’ ones. Note, however, that the error bars for *p̅*_neutralization_ values are about 5% and close-lying salt cations are assigned to ‘end-cap’/‘cylindrical’ domains using a hard cutoff boundary scheme.

Micelle surfaces are thus stabilized by electrostatic networks among CAPB ammonium moieties, SDS anionic beads, and salt cations. The main features of these networks are sensitive to the strength of the interactions between salt cations and surfactant charged moieties. For instance, strong salt cation/SDS anion head interactions as modeled by force field **R4** yield disklike micelles for *c*_salt_ = 1 M, micelles whose central main domain only comprises SDS chains all neutralized by salt cations, whereas CAPB chains are all located at their lateral ‘rim’; see Figure 7b. Force fields corresponding to weaker salt cation/SDS anionic head interactions (**R1**–**R3**) yield salt cation/SDS/CAPB networks that are overall homogeneous in compositions regardless of the micelle sub domains.

### 3.5 End-Cap/Cylindrical Equilibrium

Within the pseudophase approach framework,^58,60,87^ the proportions of surfactant chains in specific micelle subdomains (like ‘end-cap’ or ‘cylindrical’) are a quantity computed globally for a solution, i.e., there is, *a priori*, no relation between these proportions and the micelles to which the chains belong. Because of the micelle morphologies along our final MD segments, we will focus the forthcoming discussions on ‘end-cap’ and ‘cylindrical’ chains and we will neglect ‘junction’ chains that are located in micelle concave sections (as those identified in ‘giant’ micelles for *c*_salt_ = 1.7 M).

The generalization of the pseudophase framework to multicomponent solutions has been proposed.^30^ However, for our mixed micellar systems, it is far from obvious to define the surface area for CAPB chains as their ending carboxylate moieties largely lie above the micelle surface defined by SDS anionic heads, by about 6–7 Å (which corresponds to about 25% of the CAPB chain length *l̅*_CAPB_). Ending CAPB carboxylate beads may thus be considered as highly (not fully) hydrated. As such, they cannot be reasonably considered as lying at micelle surfaces (even if they define the upper bound of the micelle corona). We thus propose an alternative route to investigate ‘end-cap’/‘cylindrical’ chain equilibria in our mixed solutions by taking into account only SDS chains.

We estimate the mean proportions *p̅*_*ec*_ and *p̅*_*cyl*_ of SDS chains from the projections *Z*_anionic_^1^ of SDS anionic beads along the micelle inertia axis **Z** ^1^ and according to the conditions denoted as **1** and **2** in Section 3.3. We plot in Figure 9a the evolutions of the instantaneous ratios *r*_e_ = *p*_cyl_/*p*_ec_ computed along the final MD segments for all identified micelles and *c*_salt_ ≤ 1 M. These ratios fluctuate around mean constant values *r̅*_e_. Thus, an equilibrium exists between ‘cylindrical’ and ‘end-cap’ SDS chains in our simulated solutions. Δ^0^μ is the difference between the standard reference potentials μ_cyl_^0^ and μ_ec_^0^ of the latter chains computed from the mean ratios *r̅*_*e*_ according to Boltzmann’s relation

5 $$\Delta \mu^{0}=\mu_{cyl}^{0}-\mu_{ec}^{0}=RTln\left(\frac{{\mathrm{p̅}}_{cyl}}{{\mathrm{p̅}}_{ec}}\right)$$

For force field **R3**, Δμ^0^ linearly increases until zero at X = 2.7 (*c*_salt_ = 1 M). Then, it obeys a new regime tied to the presence of elongated micelles (which yields *p̅*_cyl_ > *p̅*_ec_), with the Δμ^0^ value increasing, *a priori*, at a much higher rate. Note that even if our protocol is, *a priori*, not suited for *c*_salt_ = 1.3–1.7 M we estimate *r*_e_ to amount to 2.65 and 3.22 (see Figure S8; Δμ^0^ of 0.58 and 0.70 kcal mol^–1^). We may relate this new Δμ^0^ regime to the start of the viscosity peak of the salt curve. For force fields **R1** and **R2** (corresponding to weaker salt cation/SDS head interactions than for **R3**), Δμ^0^ is still negative for *c*_salt_ = 1 M (it amounts to −0.21 and −0.16 kcal mol^–1^, respectively; see Figure S6). The transition toward ‘infinite’ wormlike micelles is thus expected to occur at a higher X value for these force fields compared to **R3**, as well as the viscosity peak of the salt curve.

**Figure 9:**
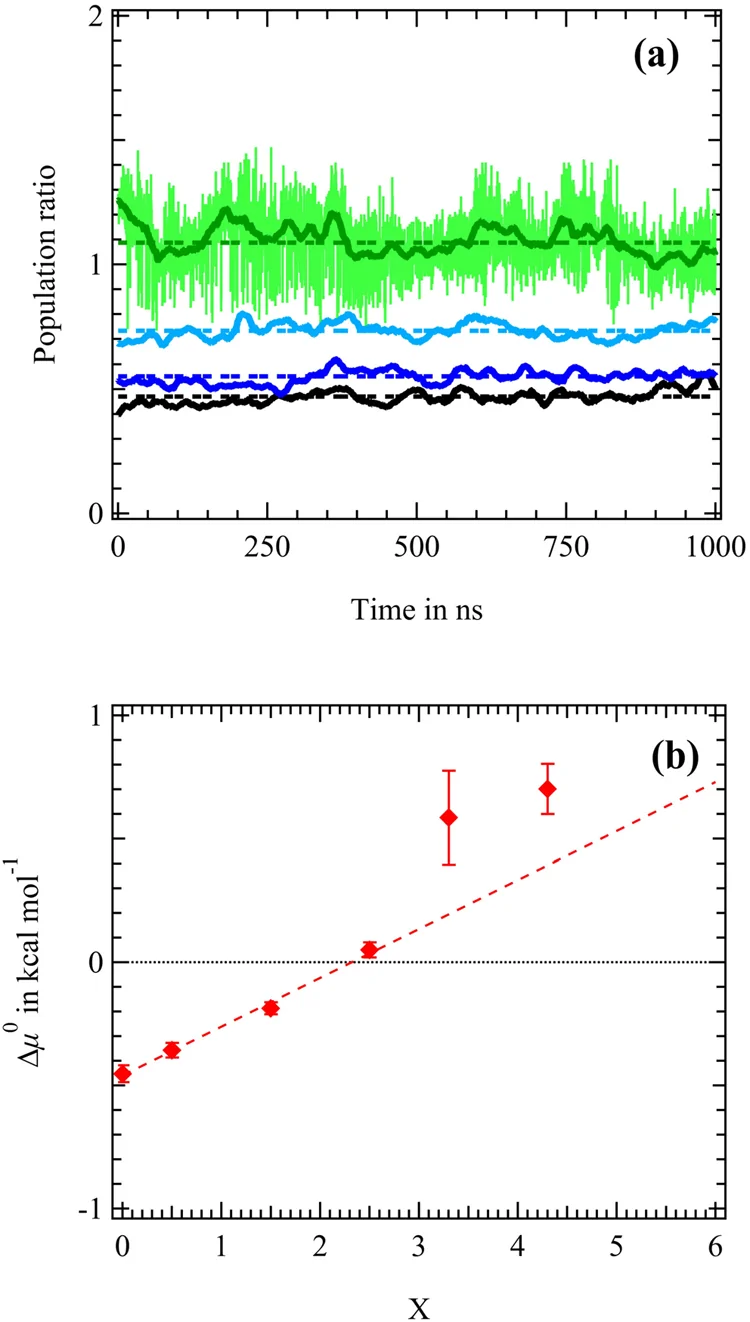
(a) Ratios of ‘cylindrical’ vs ‘end-cap’ numbers of SDS chains in 3:1 mixed SDS/CAPB solutions for *c*_salt_ = 0.0, 0.2, 0.6, and 1.0 M (in black, dark blue, light blue, and green, respectively) as a function of time along final MD segments (data for *c*_salt_ = 1.3 and 1.7 M are provided in Figure S7). Bold lines: 20 windows running averages. Thin lines: raw data for *c*_salt_ = 1.0 M. (b) Free energy difference Δμ^0^ between the ‘cylindrical’ and ‘end-cap’ standard reference potentials as a function of the ratio *X*. Dashed red line: linear regression fit for data corresponding to *c*_salt_ ≤ 1.0 M (Pearson’s regression coefficient: 0.997).

From the ‘infinite’ micelles occurring at *c*_salt_ = 2.5 M, we estimate the core volumes *v̅*_core_ for both SDS and CAPB chains (the molecular volume that corresponds to a full SDS chain and to the segment extending from the ammonium to the ending hydrophobic bead of CAPB). The total micelle volume is computed by considering the mean SDS anionic head distances along both the micelle inertia axes orthogonal to **Z** ^1^ and by assuming the main micelle section as fully rectangular or as a rectangle ended by two halves of a circle. This yields *v̅*_core_ to be about 483 ± 11 Å^3^. Assuming the full ‘infinite” micelle surfaces to comprise only SDS anionic beads (*i.e*., we implicitly account for CAPB chains), we estimate the apparent surface area *ã*_*cyl*_ to amount to 54 ± 5 Å^2^. By taking into account the mean distances *R*_bead_^*m*^ of SDS anionic beads in spheroidal micelles corresponding to *c*_salt_ = 1 M, we then get an apparent surface area *ã*_*ec*_ = 100 ± 6 Å^2^. The ratio of the values *ã*_*cyl*_ and *ã*_*ec*_ agrees with those used in ref 23 for pure SDS solutions. Our *ã*_cyl_ and *ã*_ec_ values yield the expected value (1/3) of the packing parameter *P*_0_ for spheroidal micelles. For the ‘infinite’ wormlike micelle, *P*_0_ is larger than its ideal value 1/2 (it amounts to about 3/5). This may result from the oval tubular morphology of our ‘infinite’ micelles.

Our approach based on implicitly accounting for CAPB chains is supported by the experiment as the properties of SDS/CAPB solutions, including their sphere-to-rod critical concentrations and hydrodynamic diameters, are close to pure SDS solutions for CAPB fractions up to 0.3.^10^ Lastly, for weak amounts of added salt (*c*_salt_ ≤ 0.6M), a few single free surfactant chains (all SDS) are identified along our final MD segments. Their proportions relative to ‘cylindrical’ chains are 1.6, 0.08, and 0.02% for *c*_salt_ = 0.0, 0.2, and 0.6 M, respectively.

### 3.6 Shear Viscosities

Along each MD final segment, we select a few snapshots (usually 4 for *c*_salt_ ≤ 1.3 M) corresponding to different micelle sets. We then compute the shear viscosity of each of the micellar solutions corresponding to these new MD starting geometries. Examples of the shear viscosities η­(*t*) averaged over all simulation data for each salt concentration *c*_salt_ and a specific solution geometry are shown as a function of time *t* in Figure 10a. The mean shear viscosity values η̅_0_ (averaged over all the starting solution geometries) are plotted as a function of the ratio *X* in Figure 10b. The standard deviation among the mean shear viscosity values corresponding to the different solution geometries is at most about 10% for *c*_salt_ ≤ 1 M and weaker for larger concentrations.

**Figure 10:**
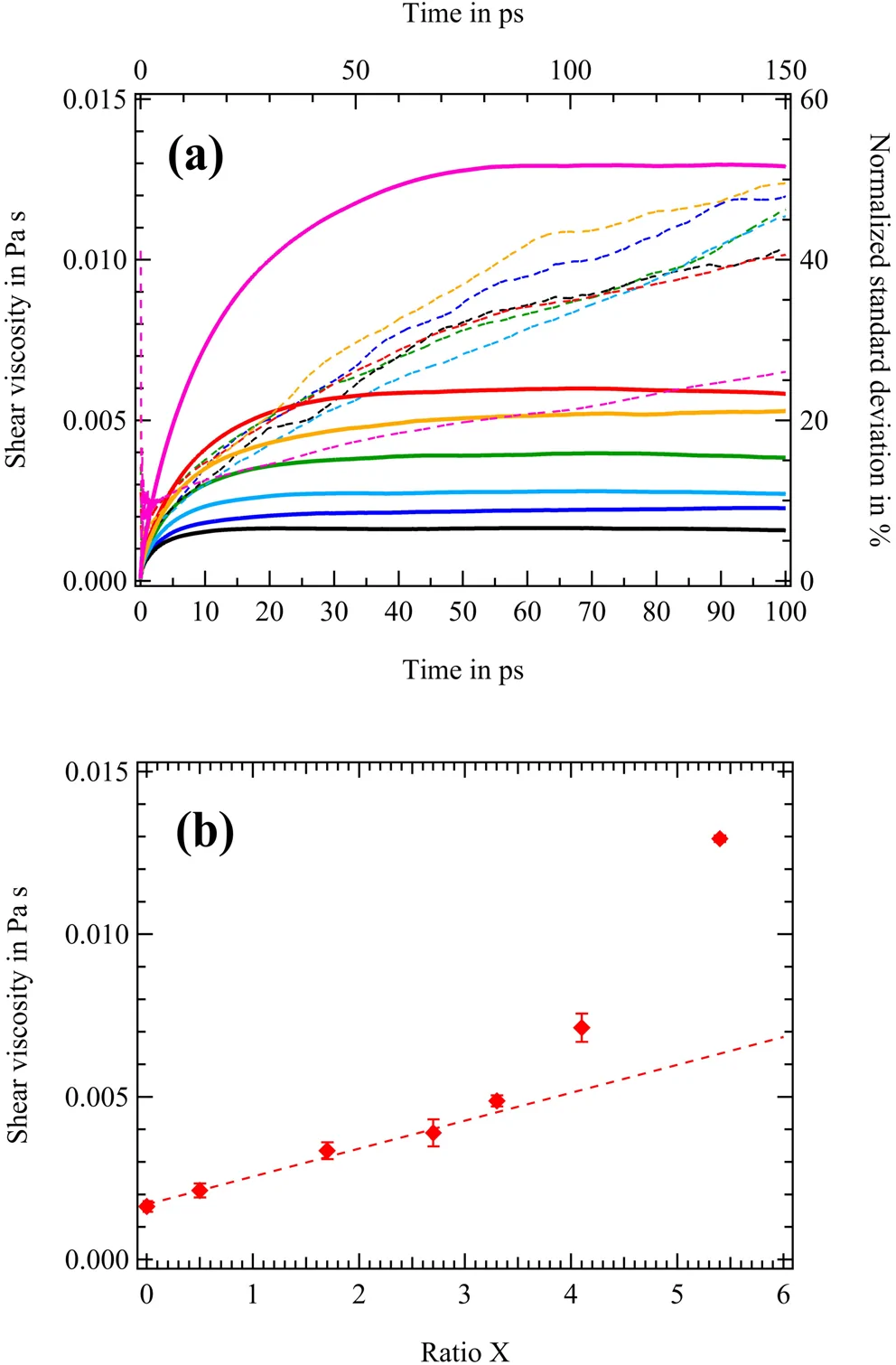
Mixed SDS/CAPB solution shear viscosities at *T* = 300 K. (a) Bold full lines (and left axis): shear viscosity η­(*t*) of micellar solutions using force field **R3** for *c*_salt_ = 0.0, 0.2, 0.6, 1.0, 1.3, 1.7, and 2.5 M as a function of time *t* (in black, dark blue, light blue, green, yellow, red, and magenta, respectively). The top axis corresponds to time *t* for 2.5 M data. Dashed thin lines (and right axis): standard deviation of η­(*t*) (in percent) among the three pressure components of the 64 independent simulations, normalized by η­(*t*) (same color definition as η­(*t*) data). (b) Mean shear viscosity η̅_0_ as a function of the ratio *X*. Dashed red line: linear regression fit for data corresponding to *c*_salt_ ≤ 1 M (Pearson’s regression coefficient: 0.992).

With no added salt, η̅_0_ is 1.6 mPa s, a value larger by 25% than the one reported for a similar solution using the most recent MARTINI force field v 3.02b with truncated long-range electrostatics (1.2 mPa s).^32^ For weak to moderate salt concentrations, η̅_0_ linearly (and slowly) increases as X increases up to 4.8 mPa s at *c*_salt_ = 1.3 M (*X* = 3.3), and then, it starts to nonlinearly and more rapidly increase until a value of 13 mPa s for the ‘infinite’ wormlike micelle solution at *c*_salt_ = 2.5 M. Hence, the disruption of the shear viscosity linear regime for weak to moderate salt concentrations to a faster increasing regime occurs at about the same *c*_salt_ value (1 M) than for the difference Δμ^0^ between ‘cylindrical’ and ‘end-cap’ reference potentials. As Δμ^0^ ≈ 0 for *c*_salt_ = 1 M, the shear viscosity linear regime for weak/moderate *c*_salt_ values is related to the predominance of spheroidal micelles in the solutions and the new regime for large salt concentrations is tied to the presence of more and more elongated micelles. This agrees with the interpretation of experimental viscosity data of SLE_1,2_ solutions at the 50 mM scale with added salt NaCl.^6^

Regarding pure alkali/halide salty aqueous solutions simulated using force field **R3**, their shear viscosity value η̅_0_ is about 1.3 ± 0.1 mPa s, regardless of *c*_salt_ (see Figure S8). This value is 30% larger than for real NaCl aqueous solutions (and for neat water). The weak ability of CG MARTINI-like simulations to estimate accurate shear viscosity data for pure and mixed anionic/zwitterionic micellar solutions has already been discussed.^32^ Nevertheless, we note here that a MARTINI-like approach is able to predict an overall constant shear viscosity value for pure salty aqueous solutions and to predict viscosity data for SDS-based micellar solutions for weak to moderate salt concentrations to be overall of the same order of magnitude as the value corresponding to neat water and/or to pure salty aqueous solutions.^25,88,89^

Regarding the shear viscosity value η̅_0_ of a mixed 3:1 SDS/CAPB solution at *c*_salt_ = 2.5 M, which, *a priori*, corresponds to the viscosity peak of the salt curve, its magnitude is much lower than expected (about 1 Pa s and above from SLES_2_ data^19,20,23^ ). High shear viscosity values of micellar solutions are inferred to arise from complex and entangled networks of long wormlike micelles (comprising thousands of surfactant chains). Here, the small size of our simulated solutions only allows us to generate a single long (‘infinite’) micelle. As we use periodic conditions, we thus simulate a parallel network of nonoverlapping ‘infinite’ micelles interacting at a long range from each other, which is far from corresponding to an entangled network.

To assess size effects on the above data, we have also simulated a 8 times larger box with three nonoverlapping ‘infinite’ micelles that are orthogonal to each other in the starting geometry (see Figure S9). This larger cell is relaxed by means of a single MD run at the 100 ns scale, along which the starting micelle network geometry is stable. From the final MD snapshot, we computed as above the shear viscosity η̅_0_ of this larger system, which amounts to 0.0152 Pa s^–1^ (see Figure S10), a value slightly larger than that for the above single ‘infinite’ micelle by about 10%. All other data discussed above regarding the single ‘infinite’ micelle are in line with those of the larger system (within the error bar). This was expected because of the nonoverlapping character of the micelle network. To simulate a realistic intricate network of (overlapping) long wormlike micelles, we need a higher level of coarse graining, for which our present simulations can provide useful data to assign its parameter.

Lastly, note that our shear viscosity values η̅_0_ for mixed SDS/CAPB solutions overall linearly increase with the mean micelle length *L̅*_*m*_ (see data of Table 2) for *c*_salt_ spanning from 1.0 to 2.5 M, whereas they are expected to be proportional to *L̅*_*m*_^3^.^90^

## 4 Conclusions

We performed Hamiltonian replica exchange (HRE) MD simulations (at least at the 2.5 μs scale) to investigate the changes in 3:1 mixed SDS/CAPB solutions upon addition of alkali/halide salts (up to a concentration of 2.5 M), by means of a coarse-grained model relying mostly on force field MARTINI version 3.02b,^49^ with, however, accounting for long-range electrostatics using a fast multipole method approach.^53^ Regarding salts, we consider four force field parameter sets to model stronger and stronger salt cation/SDS anionic head interactions. Along the final MD segments of the reference simulations (which correspond to an unaltered force field), we performed an in-depth analysis of micelle properties (like their size, morphology, and degree of neutralization) as well as the solution shear viscosities. A final MD segment is the final portion of a reference MD trajectory along which the mean micelle size is stable on a time interval of at least 1 μs. We also investigate the equilibrium among ‘end-cap’ and ‘cylindrical’ chain surfactants, as defined within the pseudophase approach.^58,60,87^

Accounting for long-range electrostatics has a strong effect on micelle sizes. With no added salt (in the presence, however, of counterions to neutralize the solution total charge), preliminary HRE simulations using the original MARTINI v 3.02b force field with truncated (for interbead distances larger than 11 Å) and untruncated electrostatic interactions show long-range effects to strongly disfavor large micelle sizes. This guides us to reassign the force field parameters handling the interactions among hydrophobic beads (modeling surfactant alkyl chains) and water beads to reproduce the lower bound of experimental pure SDS micelle sizes with no added salt (about 50 chains). We then performed all our simulations with added salts using this revised parameter set.

Using the parameter set that corresponds, *a priori*, to NaCl to model salt cation/SDS anion heads (force field **R3**), our simulations show the mixed SDS/CAPB solutions to obey two regimes. In the first regime (up to *c*_salt_ = 1 M), all the micellar solution properties (including micelle sizes and solution shear viscosities) linearly depend on the added salt concentration. For larger *c*_salt_, all these properties start to rapidly and nonlinearly diverge. Such a change in surfactant solution properties fully agrees with the experiment.^6^ In particular, for *c*_salt_ = 2.5 M, a single ‘infinite’ wormlike micelle emerges along our simulations, i.e., an elongated micelle comprising all surfactant chains and interacting at both its ends with two of its own periodic images. The appearance of an entangled network of long wormlike micelles upon addition of large enough amounts of salt is shown experimentally by cryo-TEM imaging^20^ to be tied to a strong and fast increase of the solution shear viscosity (up to a value larger by several orders of magnitude compared to adding any salt), a phenomenon called the viscosity peak of the salt curve. Even if salt curves are less documented for pure SDS and mixed SDS/CAPB solutions (in particular, as compared to SLES_1,2_ solutions), our simulations predict the viscosity peak to be located at an added salt concentration (about 2.5 M for 400 mM surfactant solutions), a priori, in line with its expected value inferred from available experimental data.

The transition of the salt curve for 3:1 mixed SDS/CAPB solutions at the 400 mM scale from the linear regime (for weak to moderate added salt concentrations) to a new regime starts at about *c*_salt_ = 1 M. Interestingly, the difference Δμ^0^ in standard potentials of SDS chains within micelle ‘end-cap’ and ‘cylindrical’ domains (according to the thermodynamics pseudophase approach) is about zero at this salt concentration. For weaker salt concentrations, we note Δμ^0^ to also linearly increase as the amount of added salt increases. The transition of our micellar solutions between the two regimes of their salt curve is tied to the increase of ‘cylindrical’ SDS chains up to be more abundant than ‘end-cap’ ones, a result also fully in line with the experiment for pure anionic surfactant solutions.^6^ Interestingly, our latter result considers CAPB chains implicitly, which is also in line with experimental data for mixed solutions when the CAPB concentration is weaker than for SDS. In this case, mixed solution properties are reported to be close to pure SDS ones.^10^ Note here that our simulations show micelle local compositions to be rather homogeneous in SDS and CAPB chains, although SDS chains are more abundant in ‘end-cap’ domains than in ‘cylindrical’ ones in elongated micelles (and conversely for CAPB). However, all our above conclusions regarding the pseudophase thermodynamical approach need to be further assessed by investigating different kinds of mixed surfactant solutions with different compositions, in particular, with CAPB in more abundance than SDS, for instance.

It is far from obvious to assign each of our CG force fields to model salt cation/SDS anionic head interactions to a specific alkali/halide salt. For instance, force field **R3**, which appears to reproduce most of the expected SDS/CAPB solution properties upon addition of NaCl salt, also predicts disklike micelles for *c*_salt_ values close to the viscosity peak of the salt curve. Such kinds of micelles are reported experimentally close to the viscosity peak, however, for the KCl salt.^20^ Moreover, we also show the overall large sensitivity of mixed SDS/CAPB solution main features to our force fields. In particular, force field **R4** corresponding to the strongest salt cation/SDS anionic head interactions yields micelle morphologies and local compositions in surfactant chains that fully differ from those of other force fields. For instance, force field **R4** predicts the presence of disklike micelles as soon as *c*_salt_ = 1 M, with SDS chains all located within micelle cores and all CAPBs within micelle ‘rims’. Only new experimental results, in particular from cryo-TEM imaging, will help us in further refining the force field parameters to model the effects of real alkali/halide salts on mixed anionic/zwitterionic surfactant solutions.

There is room to improve not only the force field used in our CG-based numerical simulation scheme but also the solvent description. In MARTINI force fields, water is treated as a nonpolar species, which is questionable when modeling the hydration of charged entities. Besides a polarizable Drude approach to improve the modeling of water,^61^ we may also consider our own orientational polarization approach.^91^ However, in all, a MARTINI-based approach (accounting long-range electrostatics) is a promising tool to investigate complex mixed anionic/zwitterionic surfactant solutions with any kind of additional compounds (including fragrance molecules, for instance). There have been attempts to propose thermodynamic models based on the pseudophase approach to predict the shear viscosity of SLE solutions when adding cosurfactants and hydrophobic (fragrance) molecules.^17,22,23,29,92^ Our numerical simulation scheme can also be used to assess and propose new data for these approaches.

## Supplementary Material







## Data Availability

Experienced academic users may request from the corresponding author a license free of charge of the code POLARIS­(MD) (including the code source). Note that POLARIS­(MD) relies on external libraries (such as FFTW and MPI ones), which prevents us to freely distribute POLARIS­(MD) binaries. Note also that here, the precision of our fast multipole method is at least equivalent to that of the smooth particle mesh Ewald approach by setting the grid interpolation size to 2 Å and by setting the B-spline interpolation to 4. Our simulations can thus also be reproduced using the latest version of the code GROMACS (https://www.gromacs.org/).

## Notes

Full list of abbreviations as well as the chain bead definition, all the force field parameters, specific data about pure alkali/halide salt aqueous solutions (PDF)
Data regarding mixed micellar solutions for all added salt concentrations (ZIP)
Raw viscosity data from simulations as well as the simulation starting geometries in a PDB format for viscosity molecular dynamics runs (ZIP)
