# Supplementary material for: Salt Effects on the Growth of Mixed Anionic and Zwitterionic Surfactant Micelles
Source: ACS Omega. 2026 Oct 16;11(42):65259–75. doi: 10.1021/acsomega.6c07682 (PMC13718207; doi:10.1021/acsomega.6c07682)
Supplement: Supplementary file 1 [file ao6c07682_si_003.pdf]

Supporting Information

Salt effects on the growth of mixed anionic and zwitterrionic surfactant micelles.

Michel Masella,[*,†] Céline Houriez,[*,‡] and Fabien Léonforté[*,¶]

†*Laboratoire de Biologie Structurale et Radiobiologie, Service de Bioénergétique, Biologie Structurale et Mécanismes, Institut de Biologie et de Technologies de Saclay, CEA Saclay, F-91191 Gif sur Yvette Cedex, France*

‡*Mines Paris, PSL University, Centre for Energy Environment Processes (CEEP), 77300 Fontainebleau, France*

¶*L'Oréal Group, Research & Innovation, 93600 Aulnay-Sous-Bois,France*

E-mail: michel.masella@cea.fr; celine.houriez@minesparis.psl.eu; fabien.leonforte@loreal.com

Contents

List of Figures **S3**

List of Tables **S5**

S1 List of abbreviations **S6**

S2 Bead types and LJ parameters **S7**

S3 Salt dissociation in neat water **S9**

S4 Salt cation/SDS anionic head Potential of Mean Force in neat water **S10**

S5 HRE scaling parameter λ^{HRE} **S10**

References **S19**

List of Figures

S1 Percentage of free ions in pure alkali/halide salty aqueous solutions using force field **R3** for salt concentrations c_{salt} spanning from 0.2 to 2.0M. Data from the final 30 ns segments of 40 ns NVT MD simulations of salty solutions comprising 8 000 water and ion beads (using the protocol detailed in the main manuscript). Free ion percentages are computed according to the numerical scheme of Ref. 2. Dashed line : best linear function $y = 100\% + a \times c_{\text{salt}}$ to reproduce the simulated data (Pearson's regression coefficient : -0.991). Error bars : standard deviations of the mean percentage of free ions. S9

S2 Temporal evolutions of the smallest (dark blue), mean (light blue) and largest (black) micelle sizes along final MD segments. Orange line (right axis) : temporal evolution of the micelle numbers. Red line : temporal evolution of the numbers of isolated chains or of surfactant assemblies comprising no more than five chains. Plots for $c_{\text{salt}} = 1$M are provided in the main manuscript. S11

S3 Micelle degrees of neutralization (red, left axis) and eccentricities (blue, right axis) for force field **R3**. Dashed red lines : mean degrees of neutralization. Plots for $c_{\text{salt}} = 1$M are provided in the main manuscript. (*) : data with no added salt by truncating long range electrostatics. S12

S4 Normalized ratios $\bar{r}^m_{\text{surfactant}}$ as a function of the micelle size for weak to moderate c_{salt} values. Black, blue and red : proportion ratios within the conic domains centered around the inertia axes $\mathbf{Z}^m$, with $m = 1, 2$ and 3, respectively. Light blue (and right axis) : micelle eccentricities. Only data from final MD segments and corresponding to micelles whose size and occurrence are larger than 20 chains and 20, respectively, are shown S13

S5 Salt cation/SDS anionic head dissociation constant K_d as a function of the ratio X. Red dashed line : best linear fit to reproduce data up to $c_{\text{salt}} = 1.3$M (Pearson's regression coefficient = 0.990). Blue dashed line : K_d converged value for $c_{\text{salt}} \geq 1.3$M. S14

S6 Ratios of 'cylindrical' *vs* 'end cap' SDS chains in 3:1 mixed SDS/CAPB solutions for $c_{\text{salt}} = 1$M as a function of time along final MD segments using force field **R1** (blue and left axis) and force field **R2** (green and right axis). Bold lines : 20-windows running averages. Thin and bold dashed lines : raw and mean data. S15

S7 20-windows running averages of 'cylindrical' *vs* 'end cap' SDS chains ratios in 3:1 mixed SDS/CAPB solutions for $c_{\text{salt}} = 1.3$ (orange) and 1.7M (red) as a function of time along final MD segments using force field **R3**. Dashed lines : mean ratio values. S16

S8 Bold full lines (and left axis) : shear viscosity $\eta(t)$ of salt aqueous solutions using force field **R3** for $c_{\text{salt}} = 0.0$, 0.2, 0.6, 1.0 and 2.0 M. Dashed thin lines: standard deviation of $\eta(t)$ (in percent) among the three pressure components of all the independent simulations, normalized by the value of $\eta(t)$. S17

S9 A representation of the final snapshot of the 'ideal' 8 times larger solution built from the relaxed 'infinite' wormlike micelle solution corresponding to $c_{\text{salt}} =$ 2.5M. Water beads are not shown. Na and Cl ions are shown in transparency mode (in green and red, respectively). The added salt concentration is again 2.5M and the cubic dimension size is 277.1 Å. The three 'infinite' micelles are orthogonal to each other and the minimal distance between them is about 40 Å. S18

S10 The shear viscosity (computed according to the protocol detailed in the main manuscript) of the 8 times larger solution with a network of three non overlapping 'infinite' micelles. S19

List of Tables

S1 LJ parameters for surfactant beads. S8

S1 List of abbreviations

SDS : Sodium Dodecyl Sulfate

CAPB : Cocamidopropyl Betaine

$SLES_n$: Sodium Dodecyl Ether Sulfate class of surfactants (with n ether groups $-O-CH_2-CH_2$)

$\bar{\eta}^0$: shear viscosity at the salt curve peak

$\bar{\eta}^0_{\mathrm{w}}$: shear viscosity of neat water

$c_{\mathrm{surfactant}}$: total surfactant concentration

c_{salt} : added salt concentration

X : ratio $c_{\mathrm{salt}}/c_{\mathrm{surfactant}}$

cryo-TEM : cryo-tomography electron microscopy

CG : coarse grained simulation

MD : Molecular Dynamics

SR : simulations with systematic truncation of long range electrostatics

LR : simulations with no truncation of long range electrostatics

HRE : Hamiltonian Replica Exchange approach

λ_{HRE} : HRE energy downscaling parameter

PES : Potential Energy Surface

P : pseudo-phase packing parameter

NVE : simulations at volume and total energy taken as constant

NVT : simulations at volume and temperature taken as constant

NPT : simulations at pressure and temperature taken as constant

N : number of NaCl added to a solution

$(\sigma_{ab}, \epsilon_{ab})$: Lennard-Jones pair of parameters for interactions between the beads a and b

FMM : Fast Multipole Method

PMF : Potential of Mean Force

d_{micelle} : inter hydrophobic bead distance to identify surfactant clusters

e : micelle eccentricity

I^m : mth moment of inertia of a micelle

Z^m : mth micelle main inertia axis

Θ^m : angle defined as $3\pi I^m/4(I^1+I^2+I^3)$

$\bar{L}_m$: mean longest micelle length

SE : micelle spheroidal-to-elongated transition

$\bar{l}_{\mathrm{SDS}}$: mean length of a single SDS chain

$\bar{l}_{\mathrm{CAPB}}$: mean length of a single CAPB chain

$\bar{n}^m_{\mathrm{SDS}}$: mean number of SDS chain in the conic domain defined by the angle Θ^m

$\bar{n}^m_{\mathrm{CAPB}}$: mean number of CAPB chain in the conic domain defined by the angle Θ^m

R^1_{max} : position of the first peak of the mean radial distribution functions of salt cations centered at SDS anionic beads

R^2_{min} : position of the first minimum after the above first peak

Q_{m} : micelle total charge without accounting for counter ions/salt cations

$\bar{p}_{\mathrm{neutralization}}$: mean degree of neutralization of a micelle by its surrounding counter ions and salt cations.

K_d : equilibrium constant corresponding to the dissociation of close lying salt cations from micelle surfaces

c_{charged} : amount of unneutralized SDS chains in solution

γ^* : product of the activity coefficients of bulk free salt cations and of unneutralized SDS chains at micelle surfaces

$\bar{p}_{ec}$ and $\bar{p}_{cyl}$: mean proportions of SDS chains in *end cap* and *cylindrical* micelle sub domains

r_e : ratio p_{cyl}/p_{ec}

μ^0_{cyl} and μ^0_{ec} : standard reference potentials of *end cap* and *cylindrical* SDS chains

$\Delta\mu^0$: difference in μ^0_{cyl} and μ^0_{ec} values

$\bar{v}_{\mathrm{core}}$: core volumes of SDS and CAPB surfactant chains

$\tilde{a}_{cyl}$ and $\tilde{a}_{ec}$: surface area of SDS anionic heads at micelle surfaces

S2 Bead types and LJ parameters

We use the following MARTINI bead definition for SDS and CAPB surfactants :

- SDS anionic head and each of its three hydrophobic beads (modeling the 12-long alkyl chain) : Q1 (*regular*) and C1 (*regular*), respectively;
- CAPB carboxylate, ammonium, amide and each of its three hydrophobic beads (modeling the CAPB 12-long alkyl chain): SQ1 (*small*) , Q1 (*regular*) , P5 (*regular*) and C1 (*regular*), respectively.

Salt anions and cations are modeled as *tiny* ionic TQ1 beads. Refined parameter sets to model salt anion/cation, salt cation/water bead and salt cation/SDS anionic head interactions are discussed in the main manuscript. The code POLARIS(MD) allows us to consider specific LJ parameters corresponding to salt cation/cation, salt cation/anion and salt anion/anion interactions as well as to SDS anionic head/salt cation interactions. In the latter case, this means that the LJ parameters used in our simulations are different than the Q1-TQ1 ones reported below (which are only used to model the CAPB ammonium/salt ion interactions). Ion/neutral bead ϵ_{ab} parameters account for the 'polarization' correction.[1]

Table S1: LJ parameters for surfactant beads.

	σ_{ab} (in nm)	ϵ_{ab} (in kJ mol^{-1})
C1 - C1	0.470	3.40
C1 - P5	0.470	2.44
C1 - Q1	0.485	2.59
C1 - SQ1	0.443	2.76
C1 - TQ1	0.404	3.31
P5 - P5	0.470	4.48
P5 - Q1	0.470	4.75
P5 - SQ1	0.430	5.74
P5 - TQ1	0.395	7.88
Q1 - Q1	0.470	3.98
Q1 - SQ1	0.438	3.72
Q1 - TQ1	0.405	4.40
SQ1 - SQ1	0.411	2.24
SQ1 - TQ1	0.385	1.25

S3 Salt dissociation in neat water

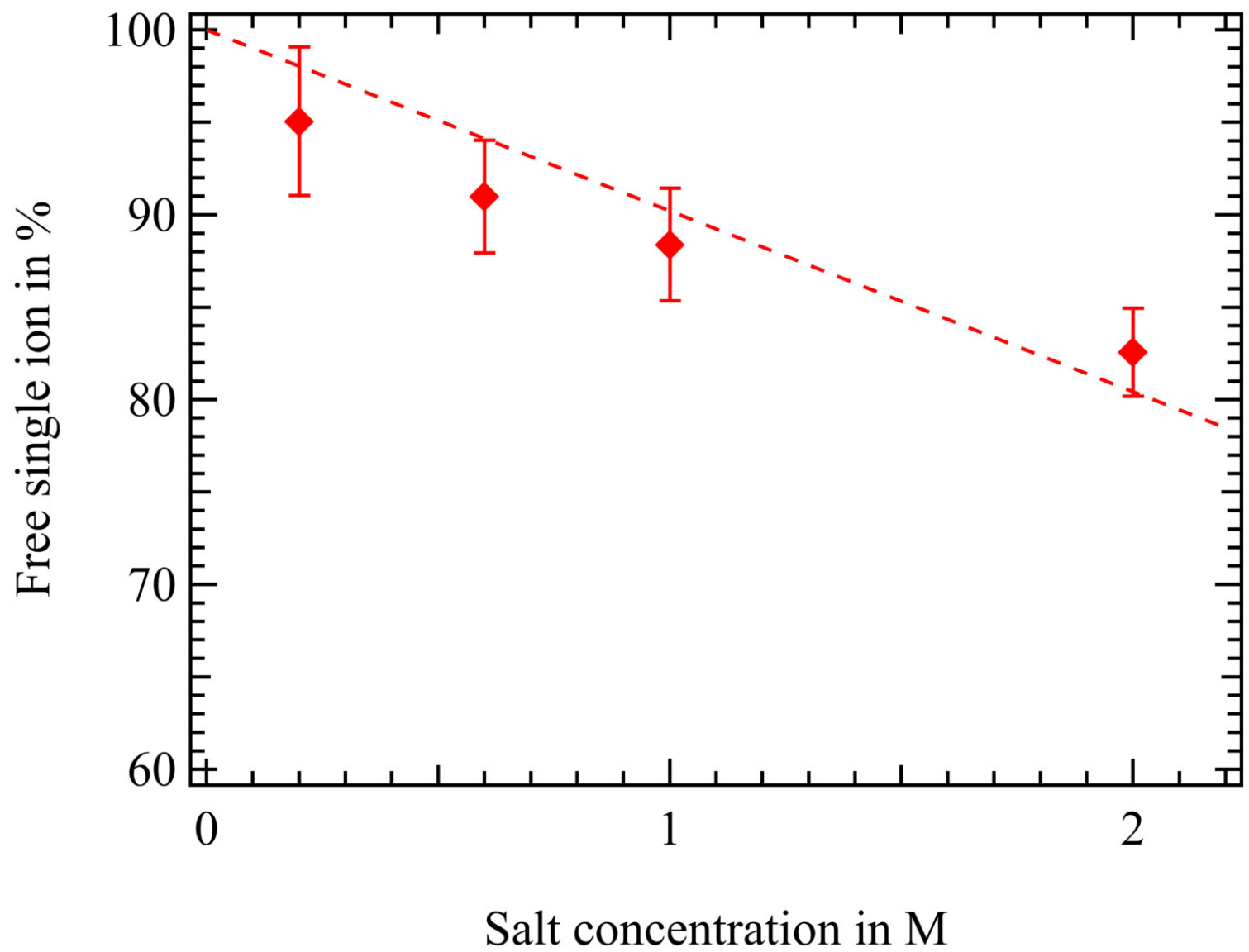

Figure S1: Percentage of free ions in pure alkali/halide salty aqueous solutions using force field **R3** for salt concentrations c_{salt} spanning from 0.2 to 2.0M. Data from the final 30 ns segments of 40 ns NVT MD simulations of salty solutions comprising 8 000 water and ion beads (using the protocol detailed in the main manuscript). Free ion percentages are computed according to the numerical scheme of Ref. 2. Dashed line : best linear function $y = 100\% + a \times c_{\text{salt}}$ to reproduce the simulated data (Pearson's regression coefficient : -0.991). Error bars : standard deviations of the mean percentage of free ions.

S4 Salt cation/SDS anionic head Potential of Mean Force in neat water

We estimate the potential of mean force, PMF, corresponding to a single salt cation/SDS chain pair dissolved in a water box by means of an umbrella sampling scheme based on MD simulations (see main manuscript for MD details). The additional energy term $U^i_{umbrella}$ added to the system total energy U is

$$U^i_{umbrella} = k_{umbrella}(R - R_i)^2. \tag{1}$$

We consider a set of target salt cation/SDS anionic head distances R^i included within 2.0 and 10.5 Å and regularly spaced by 0.5 Å. The corresponding 16 MD runs are performed at the 50 ns scale. Their last 45 ns segments are sampled each 10 ps to compute statistical data. The parameter $k_{umbrella}$ is set to 5 kcal mol^{-1} Å^{-2}. The PMF is computed from the distance R distributions using the Umbrella Integration method[3] and by accounting for the entropic component 2RT ln R. The starting structures correspond to a salt cation/SDS chain pair embedded in a cubic box whose relaxed volume is about 102.76^3 Å^3 (the box comprises 9 649 water beads). Corrections arising from the use of periodic boundary conditions to the computed PMF's are small for a solvent of high permittivity as in our simulations.[4] They are here not taken into account.

S5 HRE scaling parameter λ^{HRE}

1.00 - 0.98 -0.96 - 0.94 - 0.90 - 0.86 - 0.80 - 0.74 - 0.68 - 0.62 - 0.56 - 0.50 - 0.44 - 0.38 - 0.32 - 0.30

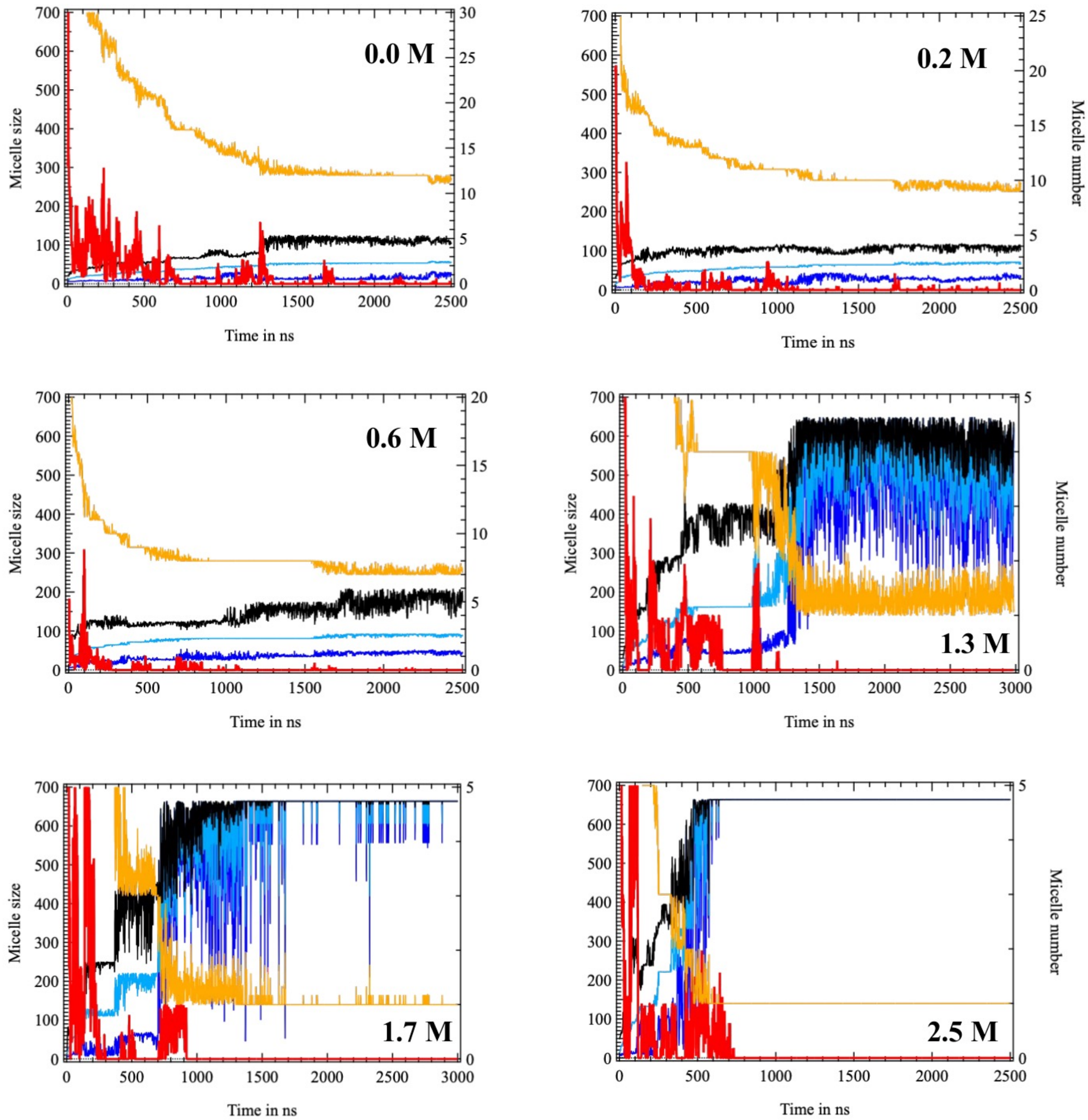

Figure S2: Temporal evolutions of the smallest (dark blue), mean (light blue) and largest (black) micelle sizes along final MD segments. Orange line (right axis) : temporal evolution of the micelle numbers. Red line : temporal evolution of the numbers of isolated chains or of surfactant assemblies comprising no more than five chains. Plots for $c_{\text{salt}} = 1\text{M}$ are provided in the main manuscript.

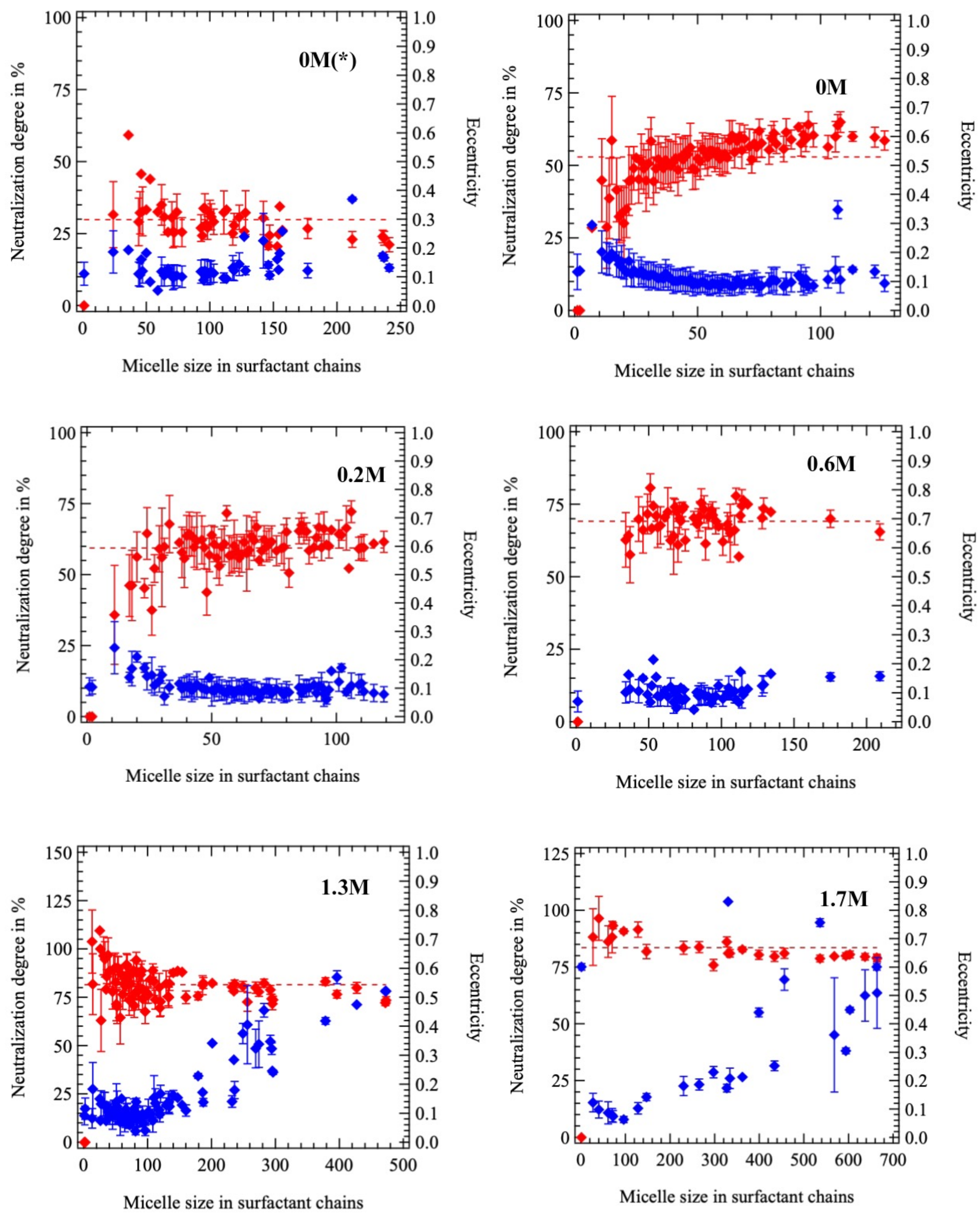

Figure S3: Micelle degrees of neutralization (red, left axis) and eccentricities (blue, right axis) for force field **R3**. Dashed red lines : mean degrees of neutralization. Plots for $c_{\mathrm{salt}} = 1\mathrm{M}$ are provided in the main manuscript. (*) : data with no added salt by truncating long range electrostatics.

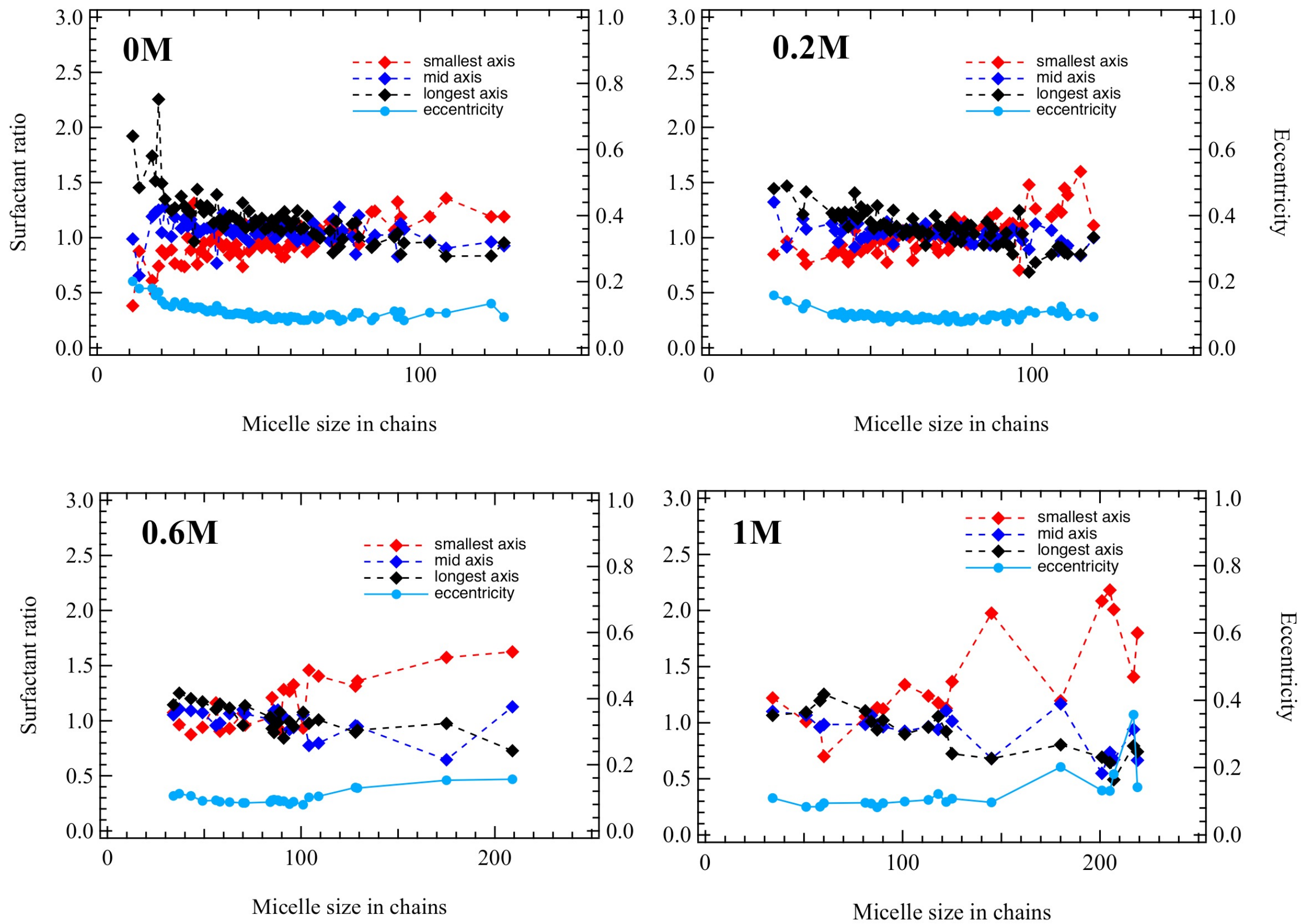

Figure S4: Normalized ratios $\bar{r}^m_{\mathrm{surfactant}}$ as a function of the micelle size for weak to moderate c_{salt} values. Black, blue and red : proportion ratios within the conic domains centered around the inertia axes $\mathbf{Z}^m$, with $m = 1, 2$ and 3, respectively. Light blue (and right axis) : micelle eccentricities. Only data from final MD segments and corresponding to micelles whose size and occurrence are larger than 20 chains and 20, respectively, are shown

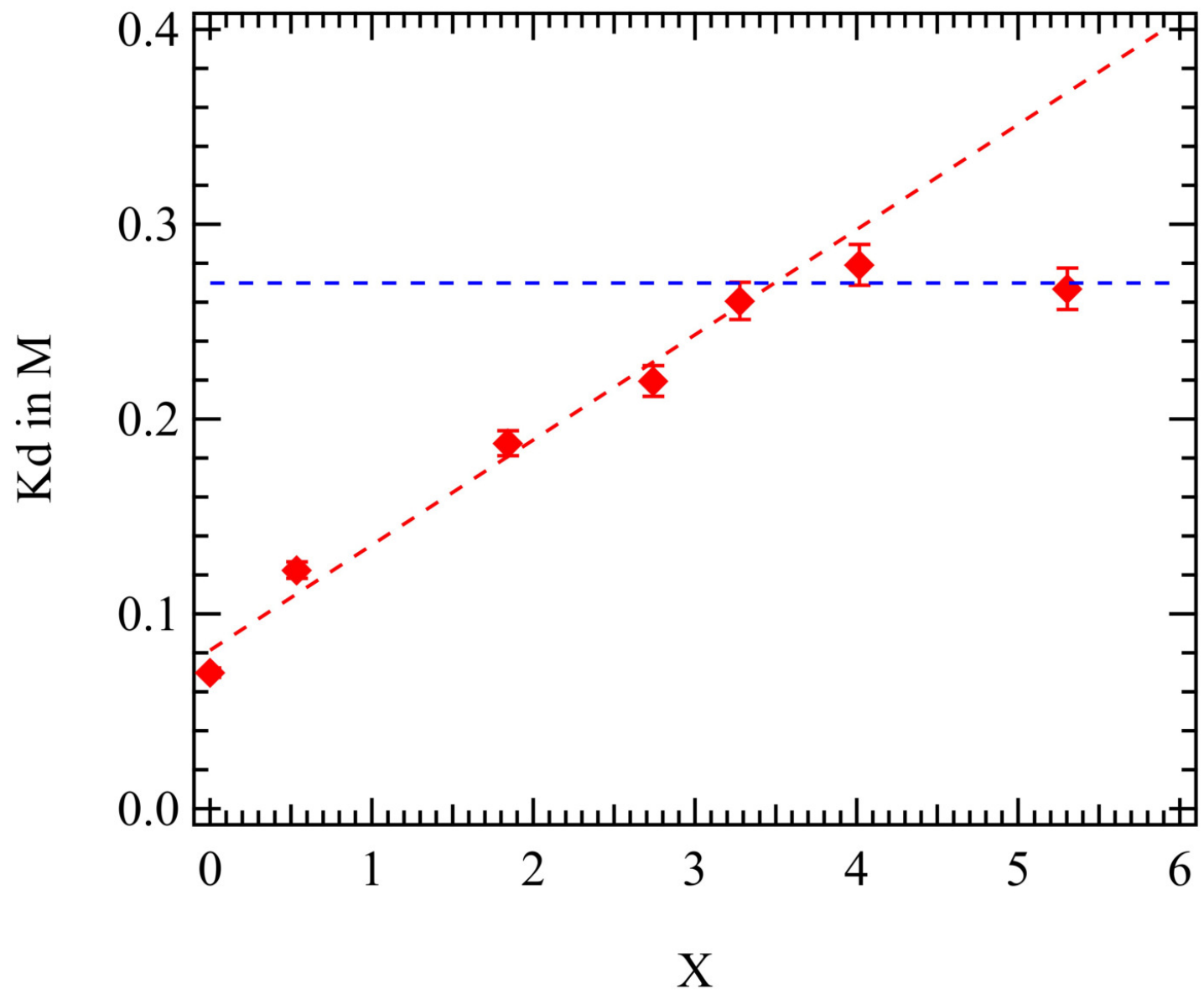

Figure S5: Salt cation/SDS anionic head dissociation constant K_d as a function of the ratio X. Red dashed line : best linear fit to reproduce data up to c_{salt} = 1.3M (Pearson's regression coefficient = 0.990). Blue dashed line : K_d converged value for $c_{\mathrm{salt}} \geq 1.3$M.

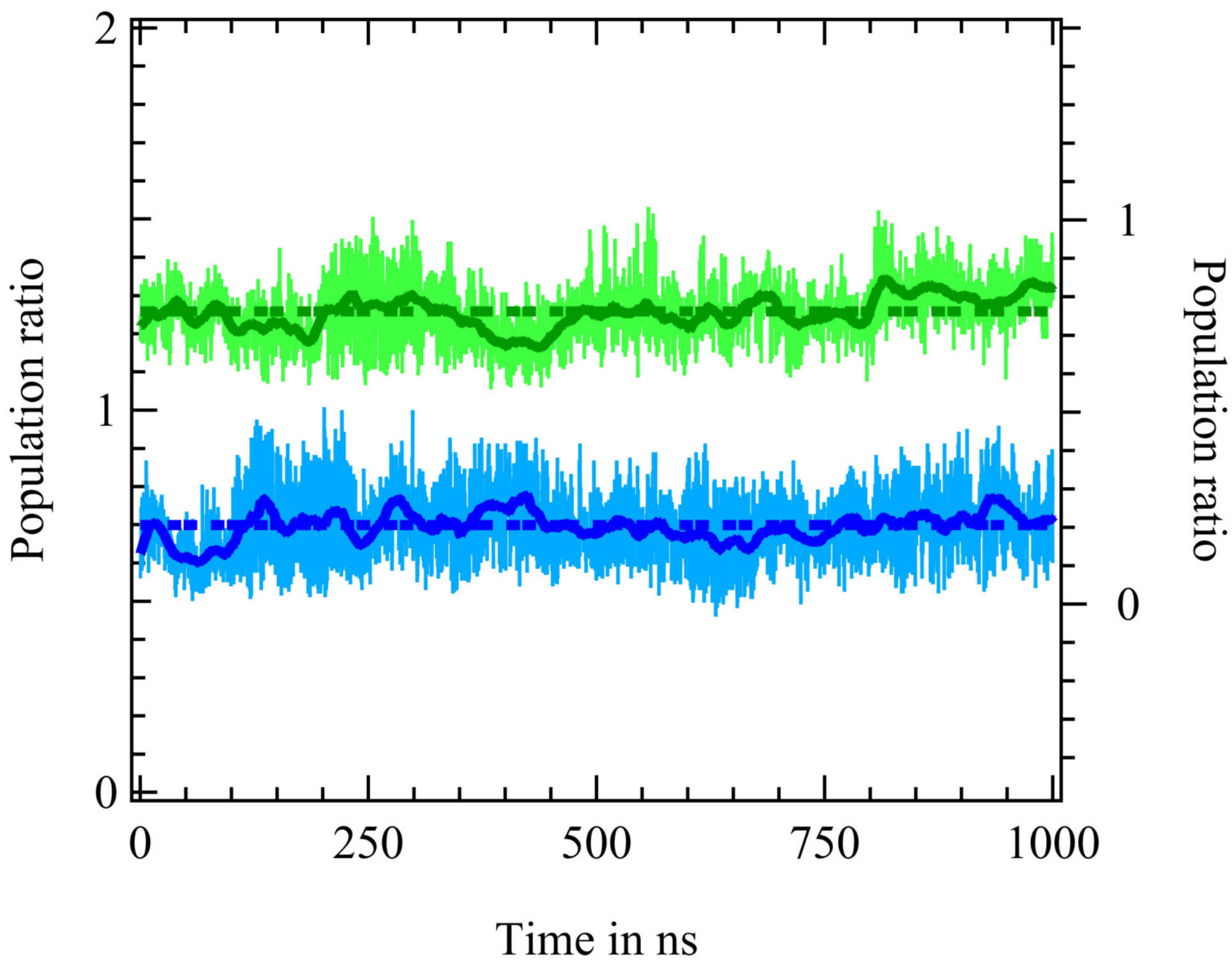

Figure S6: Ratios of 'cylindrical' *vs* 'end cap' SDS chains in 3:1 mixed SDS/CAPB solutions for $c_{\text{salt}} = 1\text{M}$ as a function of time along final MD segments using force field **R1** (blue and left axis) and force field **R2** (green and right axis). Bold lines : 20-windows running averages. Thin and bold dashed lines : raw and mean data.

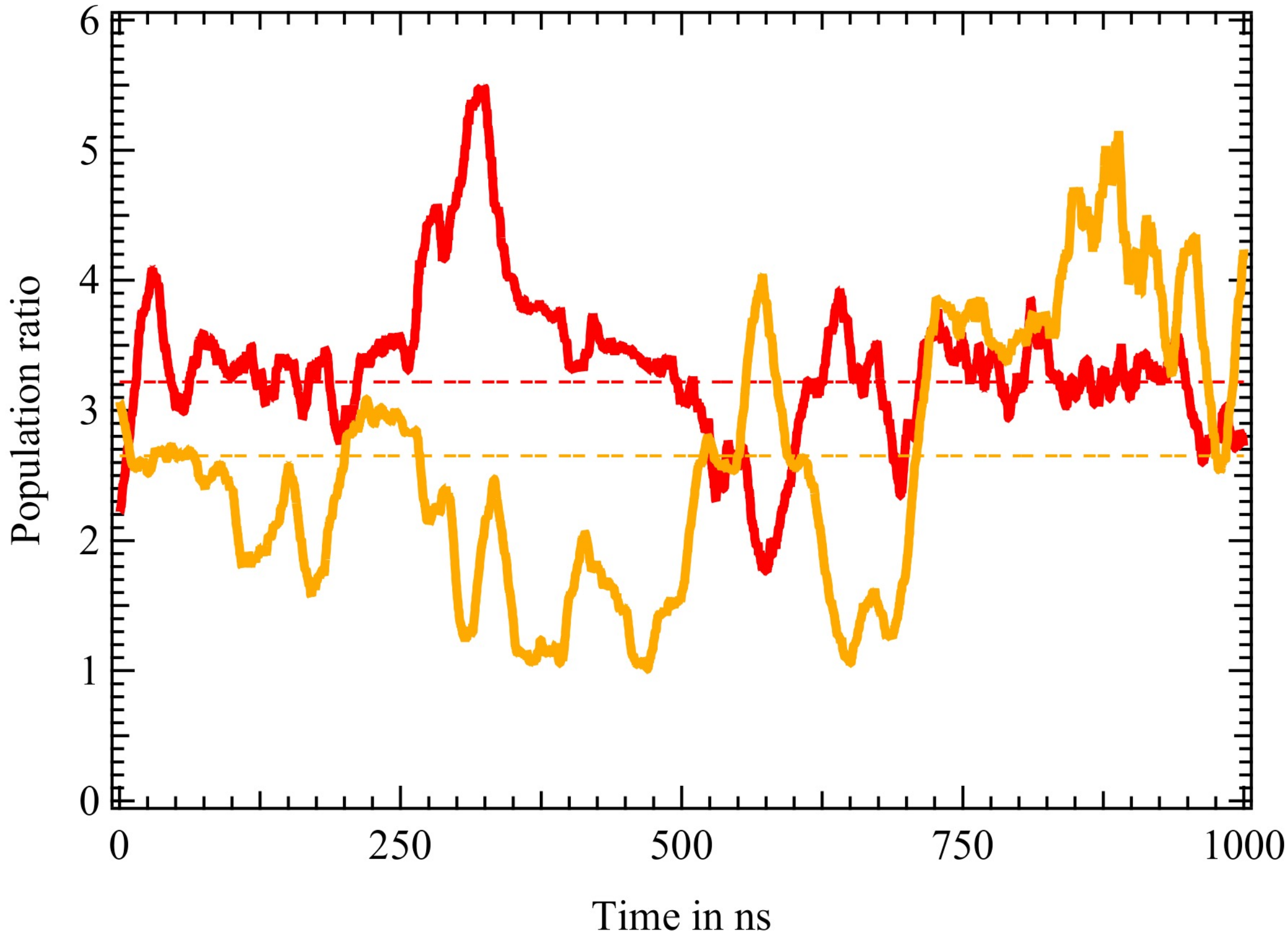

Figure S7: 20-windows running averages of 'cylindrical' *vs* 'end cap' SDS chains ratios in 3:1 mixed SDS/CAPB solutions for c_{salt} = 1.3 (orange) and 1.7M (red) as a function of time along final MD segments using force field **R3**. Dashed lines : mean ratio values.

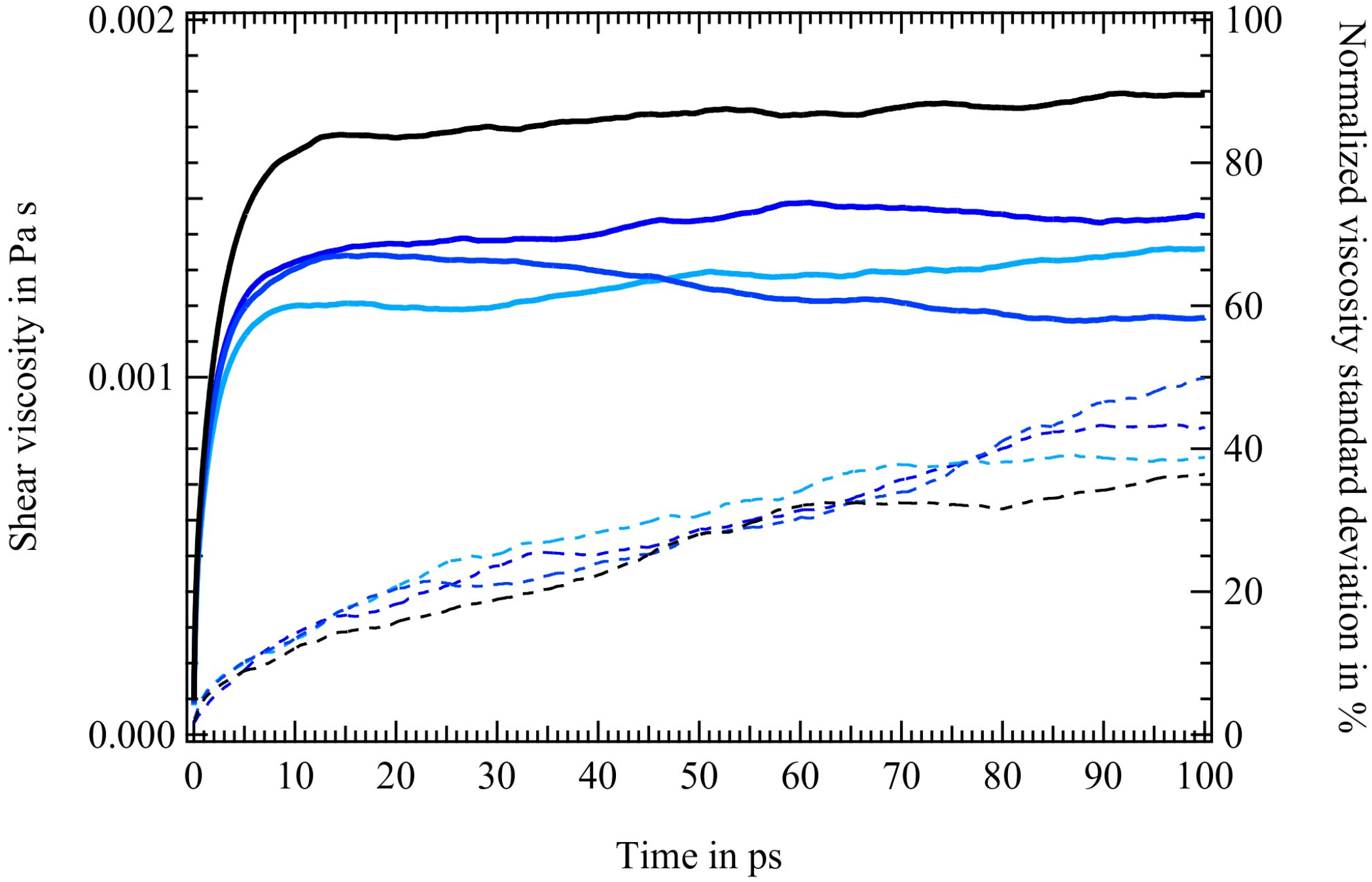

Figure S8: Bold full lines (and left axis) : shear viscosity $\eta(t)$ of salt aqueous solutions using force field **R3** for $c_{\mathrm{salt}} = 0.0$, 0.2, 0.6, 1.0 and 2.0 M. Dashed thin lines: standard deviation of $\eta(t)$ (in percent) among the three pressure components of all the independent simulations, normalized by the value of $\eta(t)$.

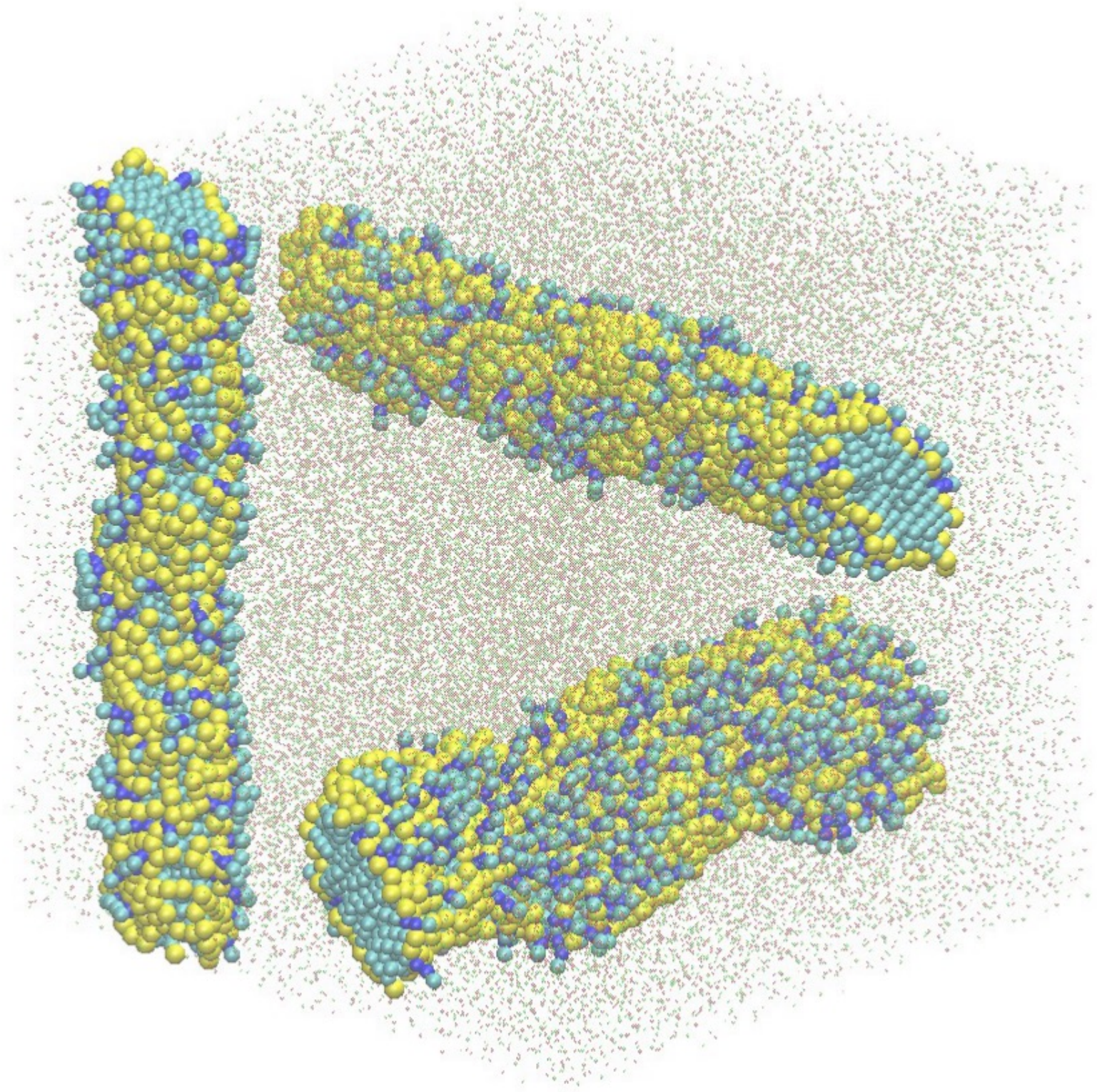

Figure S9: A representation of the final snapshot of the 'ideal' 8 times larger solution built from the relaxed 'infinite' wormlike micelle solution corresponding to c_{salt} = 2.5M. Water beads are not shown. Na and Cl ions are shown in transparency mode (in green and red, respectively). The added salt concentration is again 2.5M and the cubic dimension size is 277.1 Å. The three 'infinite' micelles are orthogonal to each other and the minimal distance between them is about 40 Å.

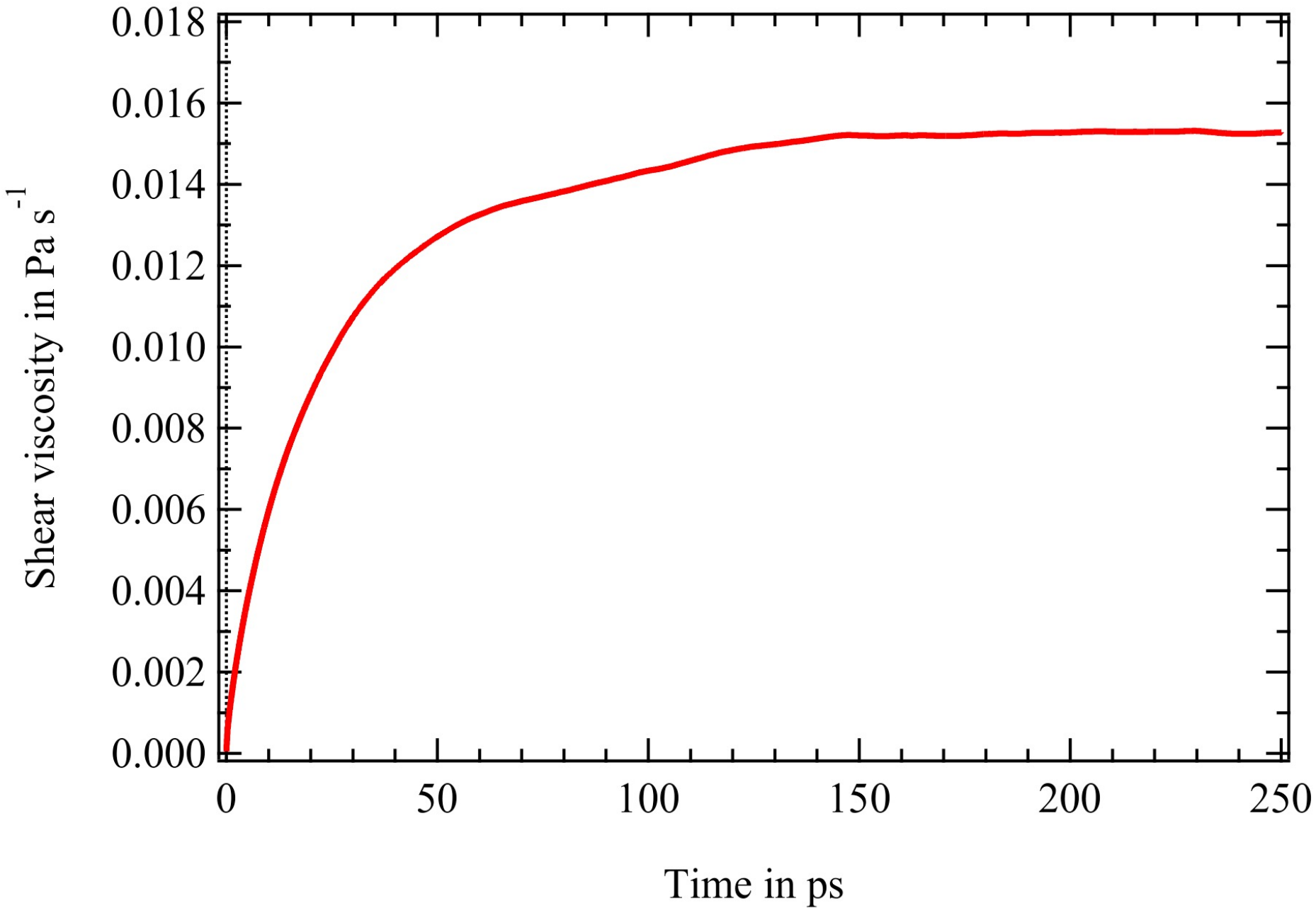

Figure S10: The shear viscosity (computed according to the protocol detailed in the main manuscript) of the 8 times larger solution with a network of three non overlapping 'infinite' micelles.

References

(1) Souza, P. C. T.; Alessandri, R.; Barnoud, J.; Thallmair, S.; Faustino, I.; Grünewald, F.; Patmanidis, I.; Abdizadeh, H.; Bruininks, B. M. H.; Wassenaar, T. A. et al. Martini 3: a General Purpose Force Field for Coarse-Grained Molecular Dynamics. *Nature Methods* **2021**, *18*, 382–388.

(2) Vallet, V.; Coles, J.; Réal, F.; Houriez, C.; Masella, M. NaCl Salts in Finite Aqueous Environments at the Fine Particle Marine Aerosol Scale. *ACS Earth and Space Chemistry* **2022**, *6*, 1612–1626.

(3) Kästner, J.; Thiel, W. Bridging the Gap Between Thermodynamic Integration and Umbrella Sampling Provides a Novel Analysis Method: "Umbrella Integration". *J. Chem. Phys.* **2005**, *123*, 144104.

(4) Hünenberger, P. H.; McCammon, J. A. Ewald Artifacts in Computer Simulations of Ionic Solvation and Ion-Ion interaction: A Continuum Electrostatics Study. *J. Chem. Phys.* **1999**, *110*, 1856–1872.
